# Combined Photothermal and mTOR‐Targeted Therapy Overcomes Immune Evasion and Enhances Checkpoint Blockade Efficacy in Metastatic Triple‐Negative Breast Cancer

**DOI:** 10.1002/advs.202513711

**Published:** 2025-11-19

**Authors:** Yujie Zhao, Jing Yu, Xin Wang, Xu Liu, Fengli Zuo, Tianyue Xu, Leyi Tang, Ling Xiong, Li Li, Huifang Li, Xiaoting Chen, Guang Yang, Jing Jing, Xiaowei Liu

**Affiliations:** ^1^ Institute of Breast Health Medicine State Key Laboratory of Biotherapy West China Hospital Sichuan University and Collaborative Innovation Center Chengdu Sichuan 610041 China; ^2^ Laboratory of pathology West China Hospital of Sichuan University Chengdu Sichuan 610041 China; ^3^ Research Core Facility West China Hospital Sichuan University Chengdu Sichuan 610041 China; ^4^ Animal Experimental Center West China Hospital Sichuan University Chengdu Sichuan 610041 China; ^5^ Breast Center West China Hospital Sichuan University Chengdu 610041 China

**Keywords:** gold nanosystem, immune “cold” tumor, immune checkpoint blockade, mTOR‐targeted therapy, photothermal, triple‐negative breast cancer (TNBC)

## Abstract

Triple‐negative breast cancer, a representative immune “cold” tumor, resists immune checkpoint blockade (ICB). A promising strategy to overcome this limitation involves combining photothermal therapy (PTT) with ICB. Here, it is demonstrated that while PTT enhances antitumor immunity by inducing immunogenic cell death (ICD), it paradoxically activates the oncogenic mTOR pathway, driving tumor immune evasion. To address this, ASPPR∩A, a mTOR inhibitor‐loaded and pH/NIR‐II‐responsive gold nanocomposite delivering localized hyperthermia and mTOR inhibition, are developed. The nanocomposite selectively targets tumor cells and efficiently converts NIR‐II light into hyperthermia upon laser irradiation. In vitro, the nanocomposite‐mediated photothermal‐mTOR dual‐therapy synergistically enhances ICD and MHC‐I antigen presentation. In murine TNBC models, this combination significantly amplifies ICD and T‐cell infiltration, and synergizes with PD‐1 blockade. Notably, this triple‐combination regimen effectively eliminates distant metastases via systemic antitumor immune response. The findings reveal the paradoxical role of PTT, establishing a photothermal‐targeted‐immune combinatorial paradigm for treating metastatic immune “cold” tumors.

## Graphical abstract

This study reveals that photothermal therapy, while inducing immunogenic cell death in triple‐negative breast cancer, paradoxically activates the oncogenic mTOR pathway to drive immune evasion. To counter this, a smart nanocomposite is engineered to co‐deliver localized hyperthermia and mTOR inhibition. This dual‐therapy synergizes with PD‐1 blockade, creating a powerful combinatorial strategy against immunologically “cold” tumors.

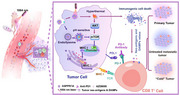

## 1 Introduction

Triple‐negative breast cancer (TNBC), accounting for 15–20% of all breast cancers, is characterized by its aggressive clinical behavior, early metastatic spread, and lack of actionable targets such as estrogen receptor (ER), progesterone receptor (PR), and human epidermal growth factor receptor 2 (HER2).^[^ ^1^, ^2^ ^]^ Patients with TNBC face a dismal prognosis, with a 5‐year survival rate of <30% for metastatic cases, underscoring the urgent need for innovative therapies.^[^ ^3^, ^4^, ^5^ ^]^ Unlike hormone receptor‐positive or HER2‐enriched subtypes, TNBC exhibits a profoundly immunosuppressive tumor microenvironment (TME), marked by deficient major histocompatibility complex class I (MHC‐I) expression, sparse cytotoxic T lymphocyte (CTL) infiltration, and enrichment of regulatory T cells (Tregs).^[^ ^6^, ^7^ ^]^ These features classify TNBC as an immunologically “cold” tumor, rendering it resistant to immune checkpoint blockade (ICB) therapies, such as anti‐PD‐1/PD‐L1 antibodies.^[^ ^8^, ^9^, ^10^ ^]^ Clinical trials, including KEYNOTE‐355, have reported objective response rates of only 18–35% for ICB monotherapy in TNBC, largely attributed to inadequate T‐cell priming and persistent immune evasion mechanisms.^[^ ^11^ ^]^ Therefore, it is necessary to develop novel strategies to agitate the immune microenvironment and improve the antitumor efficiency of ICB in TNBC.

Photothermal therapy (PTT), which employs photoresponsive nanomaterials to convert near‐infrared (NIR) light into localized hyperthermia, has emerged as a promising strategy to augment immunotherapy.^[^ ^12^, ^13^, ^14^, ^15^ ^]^ By inducing immunogenic cell death (ICD), PTT triggers the release of tumor‐associated neoantigens and damage‐associated molecular patterns (DAMPs), including calreticulin (CRT), high‐mobility group box 1 (HMGB1), and heat shock proteins, which promote dendritic cell (DC) maturation and cross‐presentation of tumor antigens to CD8^+^ T cells.^[^ ^16^, ^17^, ^18^ ^]^ Gold nanoparticles (AuNPs) with tunable surface plasmon resonance (SPR) and high photothermal conversion efficiency are particularly well‐suited for PTT. Applications of AuNPs in vitro diagnostics and cancer therapy have already received approval from the FDA.^[^ ^19^ ^]^ Given the minimally invasive, controllable, low‐risk nature of PTT combined with its robust immune‐stimulating effects, it presents substantial potential as an immunomodulatory treatment strategy. However, previous studies revealed that PTT can activate the AKT/mTOR pathway in normal cells.^[^ ^20^, ^21^ ^]^ Our results also revealed a critical paradox: while PTT enhances tumor‐associated antigen release and active immune response, it simultaneously activates the AKT/mTOR pathway of tumor cells. In TNBC, AKT/mTOR pathway hyperactivation correlates with poor prognosis, driving rapid tumor proliferation, suppressing antigen presentation, and promoting immune evasion.^[^ ^22^, ^23^ ^]^ This dual role creates a therapeutic dilemma, as PTT‐induced hyperthermia may inadvertently fuel tumor survival and immune evasion.

To reconcile this conflict, we propose a dual‐modality approach combining mTOR inhibition with PTT. mTOR inhibitors, such as AZD8055, block both mTORC1 and mTORC2 complexes, restoring MHC‐I expression and enhancing CTL infiltration. However, systemic administration of mTOR inhibitors is hampered by dose‐limiting toxicities (e.g., T cell toxicity, immunosuppression) and poor tumor accumulation. To address these limitations, we engineered nanocomposites AuNC@mSiO_2_@PAA@PEG‐iRGD∩AZD8055 (ASPPR∩A) to cooperate with PTT and mTOR targeted therapy. The nanocomposites consist of a NIR‐II‐responsive gold nanocage (AuNC) kernel, an AZD8055‐carried mesoporous silica shell, a pH‐sensitive polymer on the surface of a silica shell, and a tumor homing peptide iRGD conjugated on the PAA. This design enables: 1) pH‐dependent release of AZD8055 in the acidic endosomes/lysosomes of tumor cells, 2) NIR‐II‐triggered hyperthermia (1064 nm laser) for precise ICD induction, and 3) cooperative PTT and mTOR targeted therapy to synergically enhance antigen presentation and inhibit tumor growth. By taking advantage of ASPPR∩A‐mediated PTT and mTOR‐targeted therapy, we restored tumor antigen presentation and synergized with ICB therapy. The system provided a novel strategy for the treatment of immune “cold” tumors and metastatic tumors by integrating hyperthermia, ICB, and targeted therapies.

## 2 Results

### 2.1 PTT Induces mTOR Activation and Suppresses MHC‐I Antigen Presentation in TNBC

**Figure 1:**
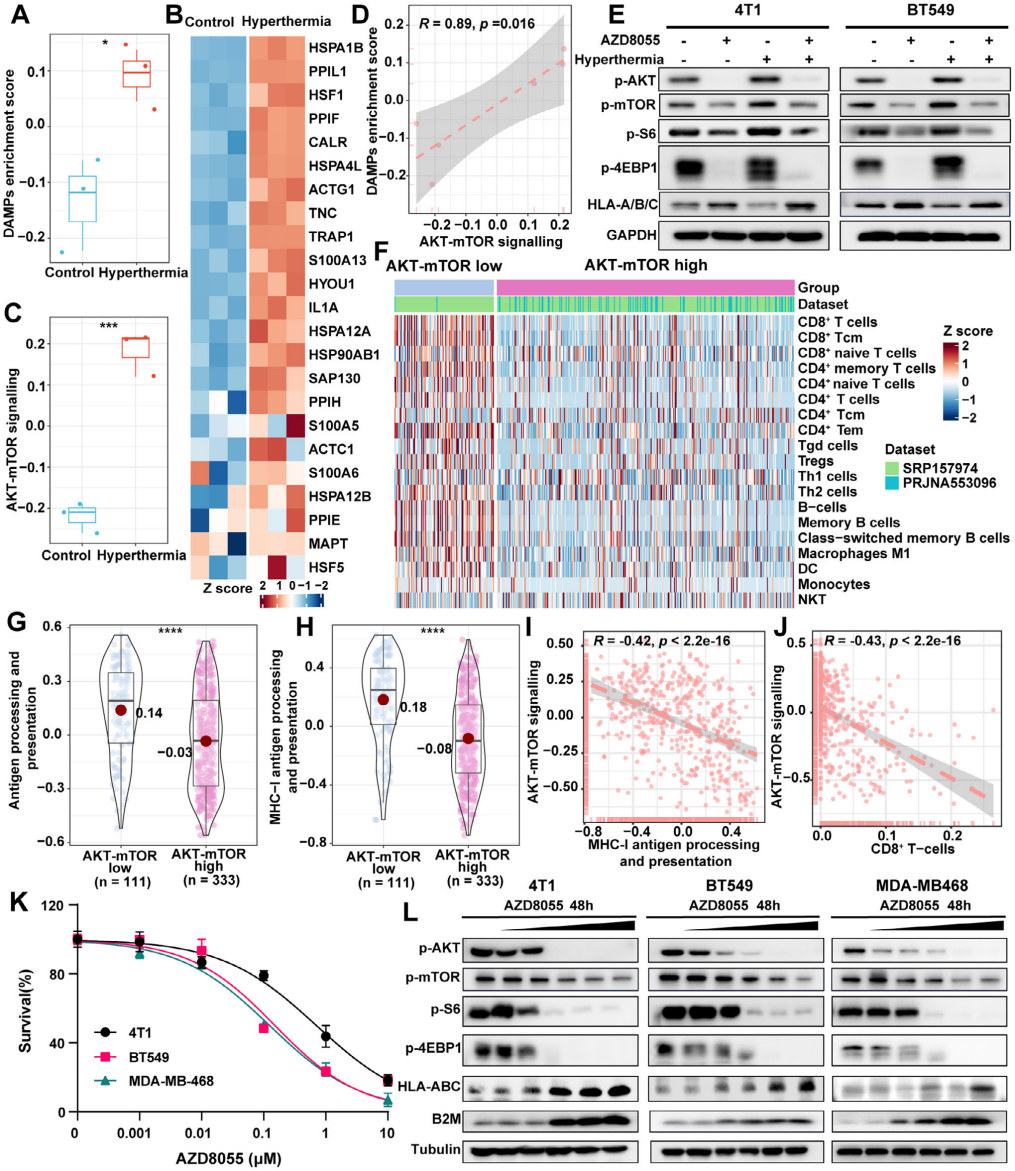
Regulation of the AKT‐mTOR pathway and MHC‐I antigen presentation in TNBC by PTT. A,B) Box plots illustrate the changes in activity of DAMPs (A) and AKT‐mTOR signaling pathway (B) in the MDA‐MB‐468 TNBC cell line before and after hyperthermia (GSE48398). Statistical testing was conducted using the Wilcoxon test. C) The expression changes of DAMPs genes in the MDA‐MB‐468 cell line before and after hyperthermia (GSE48398). D) Pearson correlation between DAMPs enrichment score and AKT‐mTOR signaling pathway (GSE48398). E) WB showed the phosphorylations of the AKT‐mTOR pathway and the MHC‐I expression in mouse TNBC cells (4T1) and human TNBC cells (BT549) treated with AZD8055 (1 µM) and hyperthermia. F) Tumor‐infiltrating immune cells were quantified using xCell, and differences between AKT‐mTOR high and AKT‐mTOR low tumors were visualized using a heatmap (Human TNBC dataset from SRP157974, PRJNA553096). G,H) Box plots illustrate the differences in GSVA enrichment scores between AKT‐mTOR high and AKT‐mTOR low tumors for antigen processing and presentation (G), MHC‐I antigen processing and presentation (H). Red dots and numerical values indicate mean values. Statistical testing was conducted using the Wilcoxon test (SRP157974, PRJNA553096). I,J) The Pearson correlation between the AKT‐mTOR signaling pathway and the MHCI antigen processing and presentation pathway (I) and the abundance of tumor‐infiltrating CD8^+^ T cells (J), respectively (SRP157974, PRJNA553096). K) The survival of 4T1, BT549, and MDA‐MB‐468 treated with different concentrations (0, 0.001, 0.01, 0.1, 1, 10 µM) of AZD8055 for 72 h. L)The expression of AKT‐mTOR pathway and MHC‐I of three cell lines (4T1, BT549, MDA‐MB‐468) treated with different concentrations (0, 0.001, 0.01, 0.1, 1, 10 µM) of AZD8055 for 48 h was analyzed by western blot. Loading controls, α‐tubulin. NES, normalized enrichment score; ^*^ *p* < 0.05, ^**^ *p* < 0.01, ^***^ *p* < 0.001, ^****^ *p* < 0.0001.

PTT enhances tumor immunogenicity through ICD, yet its molecular interplay with tumor adaptation mechanisms remains elusive. To investigate the underlying molecular mechanisms of PTT on tumor adaptation response, we analyzed the transcriptomic data from TNBC cells under hyperthermia (GSE48398) and mouse fibrosarcoma under PTT treatment (GSE224908). Consistent with previous studies,^[^ ^24^, ^25^, ^26^, ^27^ ^]^ thermal stimulation enhances the expression of DAMPs, such as heat shock proteins (HSP) and CRT, and promotes T cell infiltration in vivo (**Figure** **1**A,B; Figure S1A,B, Supporting Information). Meanwhile, several stress‐related pathways, including heat shock response, p38 MAPK signaling, hypoxia, endoplasmic reticulum stress, apoptosis, and IRE1‐mediated unfolded protein response, were also upregulated under hyperthermia stimulation (Figure S1C,D, Supporting Information). However, the AKT‐mTOR signaling pathway, a canonical oncogene pathway, was most significantly activated by PTT‐mediated hyperthermia (Figure 1C; Figure S1C–F, Supporting Information). Correlation analysis further confirmed that AKT‐mTOR activation coincided with the increase in DAMP molecules (Figure 1D; Figure S1G, Supporting Information), suggesting a paradoxical coexistence of immunostimulatory and pro‐survival responses following PTT. To validate these findings, we simulated PTT‐induced hyperthermia by exposing the cells to a high‐temperature heater. The results showed that, within a physiological temperature range, hyperthermia significantly induced the phosphorylation of AKT, 4EBP1, and S6, accompanied by decreased expression of HLA‐A/B/C. In contrast, mTOR inhibitor AZD8055 effectively suppressed phosphorylation of the AKT‐mTOR pathway and enhanced HLA‐A/B/C expression (Figure 1E). These results raise concerns about the potential pitfalls of excessive AKT‐mTOR activation, which could provide survival advantages to tumor cells, undermine the therapeutic effects of PTT, and increase the risk of tumor recurrence.

In TNBC, AKT‐mTOR activation is linked to poor prognosis and immune evasion.^[^ ^28^, ^29^, ^30^ ^]^ To dissect its immune‐modulatory role, we collected 444 human TNBC transcriptome data from SRP157974 and PRJNA553096 datasets, and categorized them into AKT‐mTOR high (n = 333) and AKT‐mTOR low (n = 111) subtypes based on the activity of the pathway. Immune infiltration and functional analysis revealed that the AKT‐mTOR high subtype tumors with reduced infiltration of immune cells (Figure 1F; Figure S2A, Supporting Information). Consistently, canonical T cell marker genes (e.g., CD3D, CD8A), immune checkpoint genes (e.g., CD274, TIGIT, CTLA4, IDO1), and cytotoxicity‐related genes (e.g., PRF1, IFNG, GZMA) were expressed at lower levels in the AKT‐mTOR high subtype (Figure S2B, Supporting Information). Accordingly, the cytotoxicity score, an index reflecting immune effector gene expression^[^ ^31^ ^]^ was significantly lower in AKT‐mTOR high tumors compared to AKT‐mTOR low tumors (Figure S2C, Supporting Information). These results collectively indicate that activation of the AKT‐mTOR pathway is correlated with impaired immune infiltration, consistent with the characteristics of an immune “cold” tumor subtype. Gene co‐expression modules and KEGG analysis highlighted marked downregulation of antigen processing and presentation pathways in AKT‐mTOR high tumors, particularly MHC‐I‐related molecules (Figure 1G,H; Figure S2D,E, Supporting Information), implying a mechanistic link between AKT‐mTOR hyperactivity and immune evasion. Strikingly, negative correlations emerged between AKT‐mTOR activity and both MHC‐I antigen presentation scores (r = −0.42, P < 0.001; Figure 1I) and intratumoral CD8^+^ T‐cell infiltration (r = −0.43, P < 0.01; Figure 1J), suggesting that AKT‐mTOR signaling drives the suppression of antitumor immunity through inhibition of MHC‐I. Our in vitro studies demonstrated that the AKT‐mTOR pathway inhibitor AZD8055, a dual‐target inhibitor of mTORC1 and mTORC2, effectively inhibited the proliferation of TNBC cell lines (Figure 1K). This inhibition downregulated the phosphorylation of AKT, mTOR, S6, and 4EBP1 in a dose‐dependent manner (Figure 1L). Conversely, treatment with the mTOR inhibitor significantly increased the MHC‐I molecules, including HLA‐A/B/C and β2M. These findings suggest that mTOR inhibition could serve as a strategy to enhance antigen presentation and synergize with PTT‐induced immunogenicity.

### 2.2 Development of pH‐Responsive NIR‐II Light‐Activated Gold Nanoparticle Drug Delivery System

**Figure 2:**
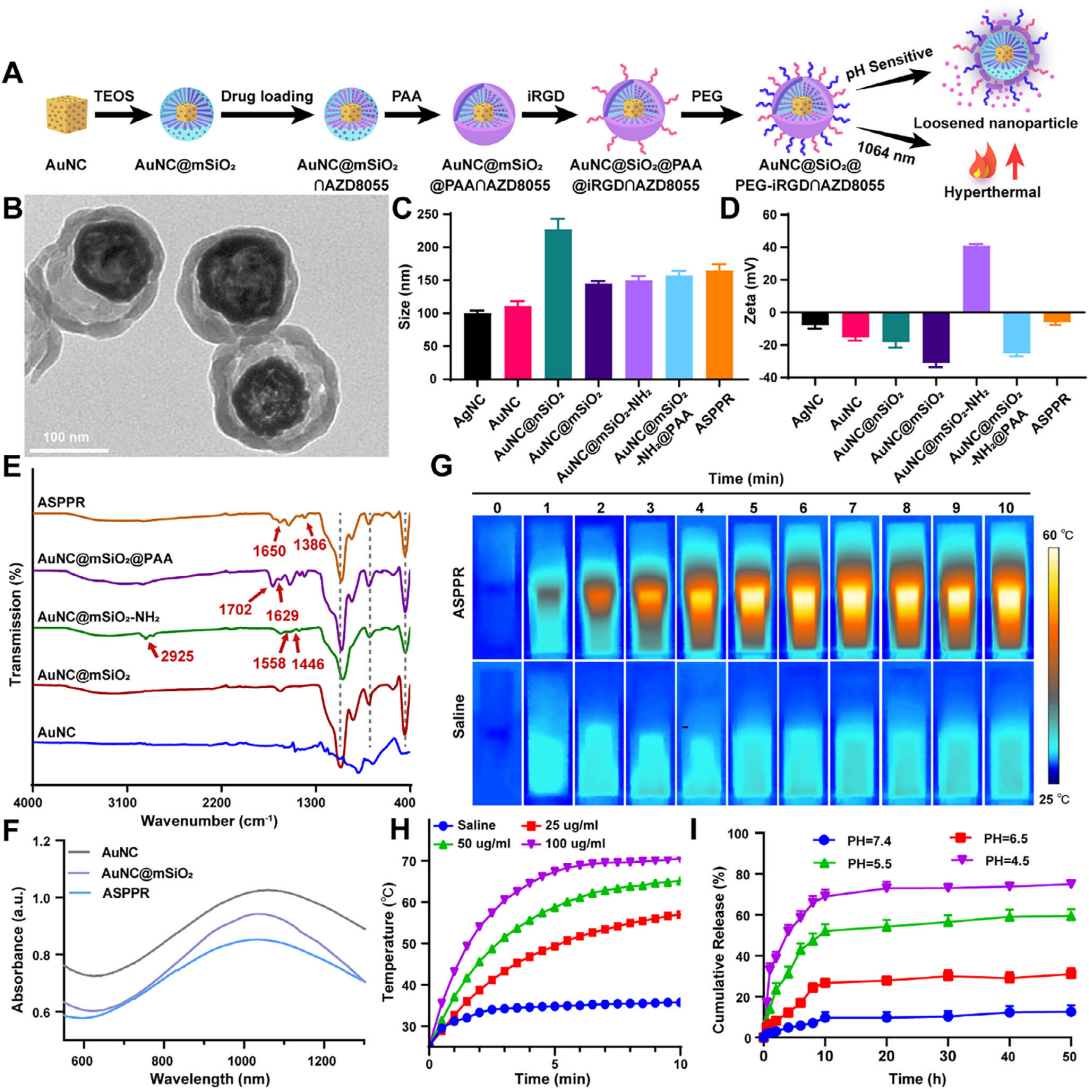
Synthesis and characterization of pH and photothermal‐responsive on‐demand controlled release nanocomposite ASPPR. A) A scheme showing the synthesis route of ASPPR, drug loading, and release. B) TEM image of ASPPR nanocomposite. Scale bars, 100 nm. C,D) Size (C) and Zeta potential (D) of AgNc, AuNc, AuNc@nSiO_2_, AuNc@mSiO_2_, AuNc@mSiO_2_‐NH_2_, AuNc@mSiO_2_@PAA, and AuNc@mSiO_2_@PAA@PEG‐iRGD (abbreviated as ASPPR), detected by nanoparticles tracking analysis. Data represented mean ± SD (n = 3). E) The FTIR spectra of AuNC, AuNC@mSiO_2_, AuNC@mSiO_2_‐NH_2_, AuNC@mSiO_2_@PAA, and ASPPR. The short gray line represents the characteristic peak of SiO_2_. F) UV–vis absorption spectra of AuNC, AuNC@mSiO_2_, and ASPPR. G) Infrared thermal images of ASPPR dispersions (50 µg mL^−1^) and saline control under 1064 nm laser irradiation (1 W cm^−^^2^) for 10 min. H) Temperature–time curves of ASPPR at different concentrations (25, 50, and 100 µg mL^−1^) compared with saline control under 1064 nm laser irradiation (1 W cm^−^^2^) for 10 min. I) Release profile of ASPPR characterized under gradient pH values. Data represent mean ± SD (n = 3).

Given the critical role of the mTOR signaling pathway in antigen presentation and immune suppression in TNBC, as well as the potential risk of mTOR activation by PTT interfering with immune responses, we designed an innovative gold nanoparticle‐based drug delivery system to optimize therapeutic outcomes. This system features pH‐responsive controlled release and can induce localized hyperthermia under NIR‐II laser irradiation (**Figure** **2**A). It not only enables the precise delivery of the mTOR inhibitor AZD8055, effectively mitigating the adverse effects of mTOR pathway activation on immune suppression, but also enhances antitumor efficacy through photothermal effects.

First, monodisperse gold nanocages (AuNCs) with an absorption peak at 1064 nm and a diameter of 110.93 nm were synthesized via the galvanic replacement reaction between silver nanocubes and chloroauric acid (HAuCl_4_) (Figure S3A–D, Supporting Information). A dense silica layer was then coated on the AuNCs via the sol‐gel method (Figure S3E, Supporting Information), and selective etching assisted by a cationic surfactant converted this layer into a mesoporous shell ≈20 nm thick. The resulting AuNC@mSiO_2_ nanocomposite exhibited a specific surface area of 56.36 m^2^ g^−1^ and a pore size of 3.75 nm (Figure S3F,G, Supporting Information). After introducing amino groups onto the AuNC@mSiO_2_ surface via modification with aminopropyltriethoxysilane (APTES), the nanoparticles were loaded with AZD8055, followed by coating with the anionic polymer polyacrylic acid (PAA) through electrostatic interactions. Finally, polyethylene glycol (PEG) and iRGD peptide were grafted onto the PAA surface, yielding a core‐shell‐membrane tri‐layered structure (AuNC@mSiO_2_@PAA@PEG‐iRGD, ASPPR, Figure 2B). Each synthesis step was validated by measuring particle size and zeta potential using nanoparticle tracking analysis (ZETAVIEW) (Figure 2C,D), which showed the expected changes in size and surface potential at each stage. Fourier transform infrared spectroscopy (FTIR) confirmed the presence of the functional groups at each stage (Figure 2E). The introduction of SiO_2_ was evidenced by absorption peaks at 1051, 778, and 443 cm^−1^, corresponding to the asymmetric stretching vibration of Si─O─Si, the bending vibration of Si─O, and the symmetric stretching vibration of Si─O─Si, respectively. After APTES modification, peaks at 2925, 1558, and 1446 cm^−1^ were observed, corresponding to the symmetric stretching vibration of C─H and the bending vibration of N─H. The AuNC@mSiO_2_@PAA displayed new peaks at 1629 and 1702 cm^−1^, attributed to the asymmetric stretching vibrations of the PAA carboxyl groups. In the final ASPPR, the peak at 1650 cm^−1^ corresponds to the characteristic amide bond (O═C─NH) formation, while the peak at 1386 cm^−1^ is attributed to the characteristic absorption of the pyridine ring in the iRGD peptide. Thermogravimetric analysis (TGA) revealed that the polymer content in ASPPR was ≈6.07% (Figure S3H, Supporting Information). We also evaluated the colloidal stability of ASPPR nanoparticles in biological medium, finding negligible changes in hydrodynamic diameter and zeta potential when incubated in 10% FBS‐containing medium over 7 days, confirming good colloidal stability under physiological conditions (Figure S3I,J, Supporting Information).

UV–vis‐NIR spectroscopy revealed that ASPPR exhibits strong absorption in the 1064 nm region (Figure 2F). To evaluate the photothermal performance of ASPPR nanocomposites, we conducted irradiation experiments at a power density of 1 W cm^−^^2^, which is commonly used in such in vitro studies.^[^ ^32^, ^33^ ^]^ The results demonstrated that ASPPR effectively induced temperature increases during irradiation and exhibited significant photothermal effects (Figure 2G,H). The photothermal conversion efficiency (ƞ) of ASPPR was calculated to be 39.88% using the Roper method (Figure S4A,B and Table S1, Supporting Information). Notably, ASPPR maintained a stable thermal response even after multiple irradiations, indicating high stability of its photothermal performance (Figure S4C, Supporting Information). To assess the feasibility of this nanocarrier system, AZD8055 was loaded into ASPPR, achieving a loading capacity of 15.8%. The AZD8055 release from ASPPR exhibited exceptional pH sensitivity: at pH 7.4, 6.5, 5.5, and 4.5, the release rates were 12.62%, 31.01%, 59.39%, and 75.27%, respectively (Figure 2I). This pronounced pH‐dependent release is attributed to the pH‐sensitive charge properties of PAA. After loading the drug into the pores of AuNC@mSiO_2_‐NH_2_, the positively charged AuNC@mSiO_2_‐NH_2_ was coated with negatively charged PAA, which encapsulated the drug particles at neutral pH. Upon a decrease in pH, PAA became protonated, leading to loosening of the shell and thus promoting drug release.^[^ ^34^ ^]^

### 2.3 Tumor‐Specific Cellular Uptake and Biodistribution of ASPPR Nanocomposite

**Figure 3:**
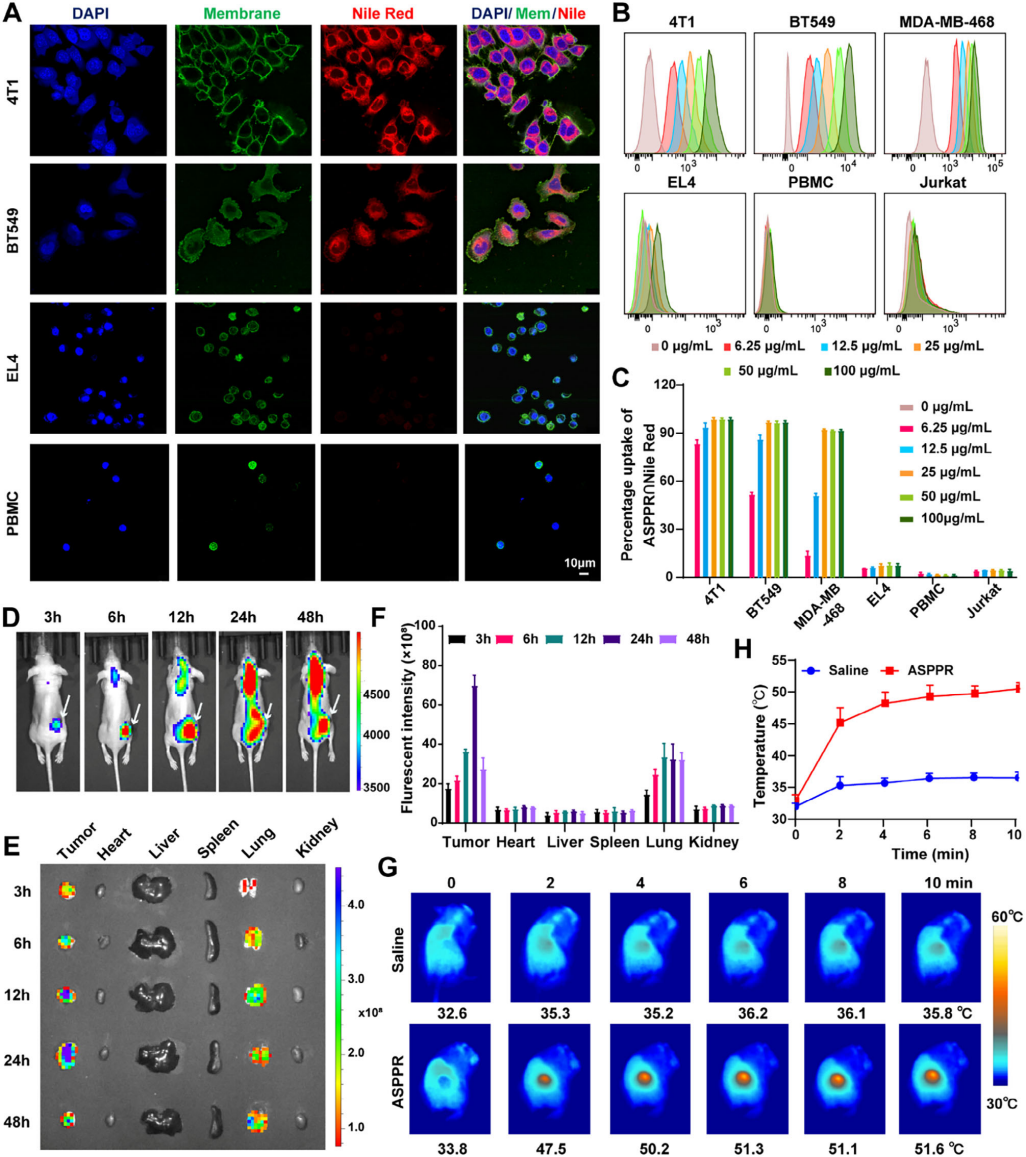
In vitro and in vivo biodistribution of ASPPR nanocomposites. A) Confocal microscopy images of the differential uptake amount of Nile Red‐labeled ASPPR (red) between TNBC cells (4T1, BT549) and lymphocytes (EL4, human PBMC). Nucleus and cell membrane were stained with DAPI (blue) and Alexa Fluor® 488 conjugated wheat germ agglutinin (green), respectively. Scale bar 10 µm. B,C) TNBC cells and lymphocytes were incubated with different concentrations(0, 6.25, 12.5, 25, 50, 100 µg mL^−1^) of Nile Red‐labeled ASPPR for 24 h. The internalizations were determined by flow cytometer (B), and the internalizations were quantified (C). The data presented mean ± SD (n = 3). D) In vivo biodistribution of Nile Red‐labeled ASPPR examined by fluorescent imaging at 3, 6, 12, 24, and 48 h post intravenous (*i.v*.) administration (the location of the xenograft tumor was indicated with the white arrows). E,F) Ex vivo imaging visualized the biodistribution of Nile Red‐labeled ASPPR in the heart, liver, spleen, lung, kidneys, as well as xenografted tumors at the indicated time points (E). The signal intensities of nanocomposites in each tissue were quantified (F). G) Changes in tumor temperatures of tumor‐bearing mice injected with ASPPR nanocomposites or saline under the 1064 nm laser irradiation. The bar heights and error bars in (F) and (H) indicate means ± SD (n = 3).

To evaluate the internalization capacity of the nanocomposites, a hydrophobic dye (Nile Red) was encapsulated into the ASPPR (denoted as ASPPR∩N) and co‐incubated with tumor cells or lymphocytes, respectively. The fluorescence signals indicating internalized nanocomposites could exclusively be observed in tumor cells rather than in lymphocytes under the fluorescence confocal microscopy (**Figure** **3**A). The internalization efficiency was further quantified by flow cytometry. The results demonstrated that the uptake efficiency of ASPPR∩N nanocomposites by tumor cells (4T1, BT549, and MDA‐MB‐468 cells) was much higher than that by lymphocytes (EL4, human PBMC, and Jurkat cells) at each concentration and time point (Figure 3B,C; Figure S5A,B, Supporting Information). After 24 h, up to 99.68% of 4T1 cells, 96.28% of BT549 cells, and 90.53% of MDA‐MB‐468 cells were positive for fluorescence signals, whereas only 7.59% of EL4 cells, 1.42% of human PBMC, and 4.65% of Jurkat cells internalized the nanocomposites (Figure S5A,B, Supporting Information). These findings indicate tumor cell‐specific uptake of ASPPR, which is primarily attributed to the lower expression levels of endocytosis‐related proteins in T cells compared to tumor cells.^[^ ^35^ ^]^

The in vivo biodistribution of ASPPR nanocomposites was evaluated by intravenously injecting the ASPPR∩N into tumor‐bearing mice and monitored the fluorescence intensity at 3, 6, 12, 24, and 48 h post‐injection. In vivo fluorescence imaging revealed that ASPPR∩N nanocomposites could be observed in tumors as early as 3 h post‐injection, with their accumulation persisting up to 48 h (Figure 3D). To further validate the in vivo biodistribution of ASPPR∩N, major organs, including the tumor, lung, heart, liver, spleen, and kidney, were dissected and examined at the specified time points. Consistent with the above results, quantitative analysis of the fluorescence signals indicated that the nanocomposites continued to accumulate in tumor tissues from 3 to 48 h due to the enhanced permeability and retention (EPR) effect (Figure 3E,F). The concentration of ASPPR∩N in tumors was significantly higher than in the heart, spleen, liver, and kidneys. Additionally, strong fluorescence signals were observed in the lungs, likely due to clearance by the reticuloendothelial system.

Encouraged by the tumor accumulation ability of the ASPPR nano‐system, we then evaluated its hyperthermia effect in vivo. Tumor‐bearing mice treated with either ASPPR nanocomposites or saline were illuminated with a 1064 nm NIR‐II laser for 2, 4, 6, 8, and 10 min, respectively. Given that higher laser power, particularly above 0.5 W cm^−^^2^, can cause skin damage at the tumor xenograft site,^[^ ^36^ ^]^ we conducted the experiments using the maximum safe irradiation power of 0.5 W cm^−^^2^, a parameter widely adopted by other studies.^[^ ^37^ ^]^ The results showed that the temperature of tumors treated with ASPPR nanocomposites was significantly higher at each time point compared to those treated with saline (Figure 3G,H). According to previous reports, the optimal temperature for PTT should range between 43 and 45 °C, a range sufficient to effectively induce necrotic cell death while being well‐tolerated by the animals.^[^ ^38^ ^]^ Consequently, a 5‐min irradiation time was selected for subsequent experiments.

### 2.4 ASPPR‐Mediated PTT and mTOR Inhibition Synergically Induce Tumor Cell Death and MHC‐I Antigen Presentation In Vitro

**Figure 4:**
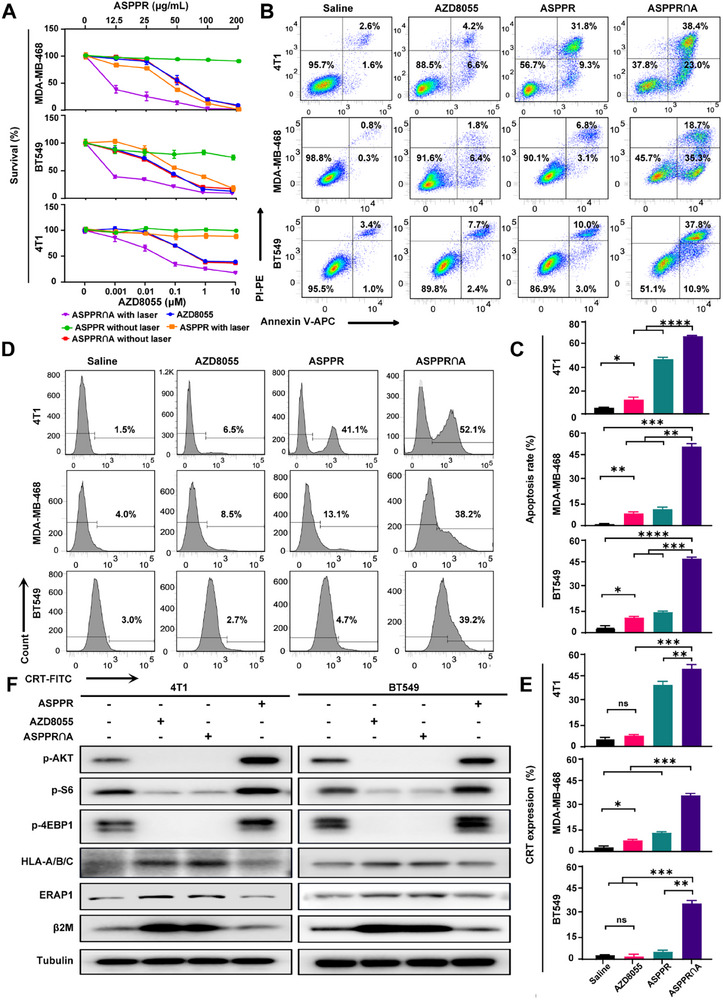
Bioactivity of ASPPR∩A nanocomposites in vitro. A) MTT survival assays of three TNBC cell lines in response to ASPPR without laser, ASPPR plus laser, AZD8055, ASPPR∩A without laser, and ASPPR∩A plus laser. B–E) TNBC cells (4T1, BT549, MDA‐MB‐468) were treated with saline, AZD8055, ASPPR with laser, or ASPPR∩A with laser. The apoptosis (B) and the expression of CRT (D) were examined by flow cytometer analysis. The apoptosis rate (C) and the CRT expression rate (E) were quantified. Statistical analysis was measured by one‐way ANOVA (n=3). ns, not significant, ^*^ *p* < 0.05, ^**^ *p* < 0.01, and ^***^ *p* < 0.001. F) The expression of PI3K/mTOR pathway and MHC‐I of three cell lines (4T1, BT549, MDA‐MB‐468) treated with 1 µM AZD8055, ASPPR plus laser, or ASPPR∩A plus laser for 24 h was analyzed by western blot. Loading controls, α‐tubulin.

We further evaluated the in vitro synergistic effects of ASPPR‐mediated PTT in combination with mTOR inhibition. Short‐term MTT assays were implemented to assess cell viability across three TNBC cell lines under varying experimental conditions: 1) AZD8055, 2) ASPPR without laser irradiation, 3) ASPPR with laser irradiation, 4) ASPPR∩A without laser irradiation, and 5) ASPPR∩A with laser irradiation (**Figure** **4**A). The results demonstrated that ASPPR without laser irradiation did not exhibit significant toxicity over a 3‐day assessment period. The cytotoxic profile of ASPPR∩A without laser irradiation was analogous to that observed in the control group AZD8055. ASPPR with laser exposure moderately inhibited cell growth, while ASPPR∩A with laser irradiation significantly suppressed cell proliferation due to the synergistic effects of PTT and mTOR inhibition. Flow cytometry was performed to analyze the apoptosis of tumor cells under ASPPR∩A nanocomposites treatment, further confirming this result. The results showed that laser‐activated ASPPR∩A nanocomposites efficiently induced tumor cell death and were significantly higher than either AZD8055 or ASPPR + laser treatment alone (Figure 4B,C). Importantly, we found that the expression of CRT, a canonical marker of ICD, on the tumor cell surface was significantly enhanced in the ASPPR∩A + laser group (Figure 4D,E). ASPPR∩A‐mediated PTT and mTOR‐targeted therapy synergistically increase CRT exposure on the tumor cell membrane. These results indicate that ASPPR∩A + laser induces the ICD, which could augment antitumor immune efficiency in vivo.

To explore the molecular mechanisms underlying this synergy, we performed western blot analysis to measure the phosphorylation levels of key AKT‐mTOR pathway components and the expression of MHC‐I‐related molecules in TNBC cells treated with ASPPR, free AZD8055, and the ASPPR∩A combination. The results showed that PTT upregulated the phosphorylation of AKT, S6, and 4EBP1, whereas AZD8055 and the ASPPR∩A combination significantly inhibited the phosphorylation of AKT, S6, 4EBP1, and mTOR. Meanwhile, the MHC‐I‐related molecules, including HLA‐A/B/C, ERAP1, and B2M, were upregulated by the AZD8055‐mediated mTOR pathway inhibition (Figure 4F). All these results demonstrated that ASPPR‐mediated PTT and mTOR inhibition not only directly inhibit tumor cell growth but also induce ICD of tumor cells and enhance MHC‐I antigen presentation.

### 2.5 ASPPR‐mTORi Synergize with anti‐PD‐1 to Inhibit Tumor Growth and Metastasis by Regulating the Anti‐Tumor Immune Response

**Figure 5:**
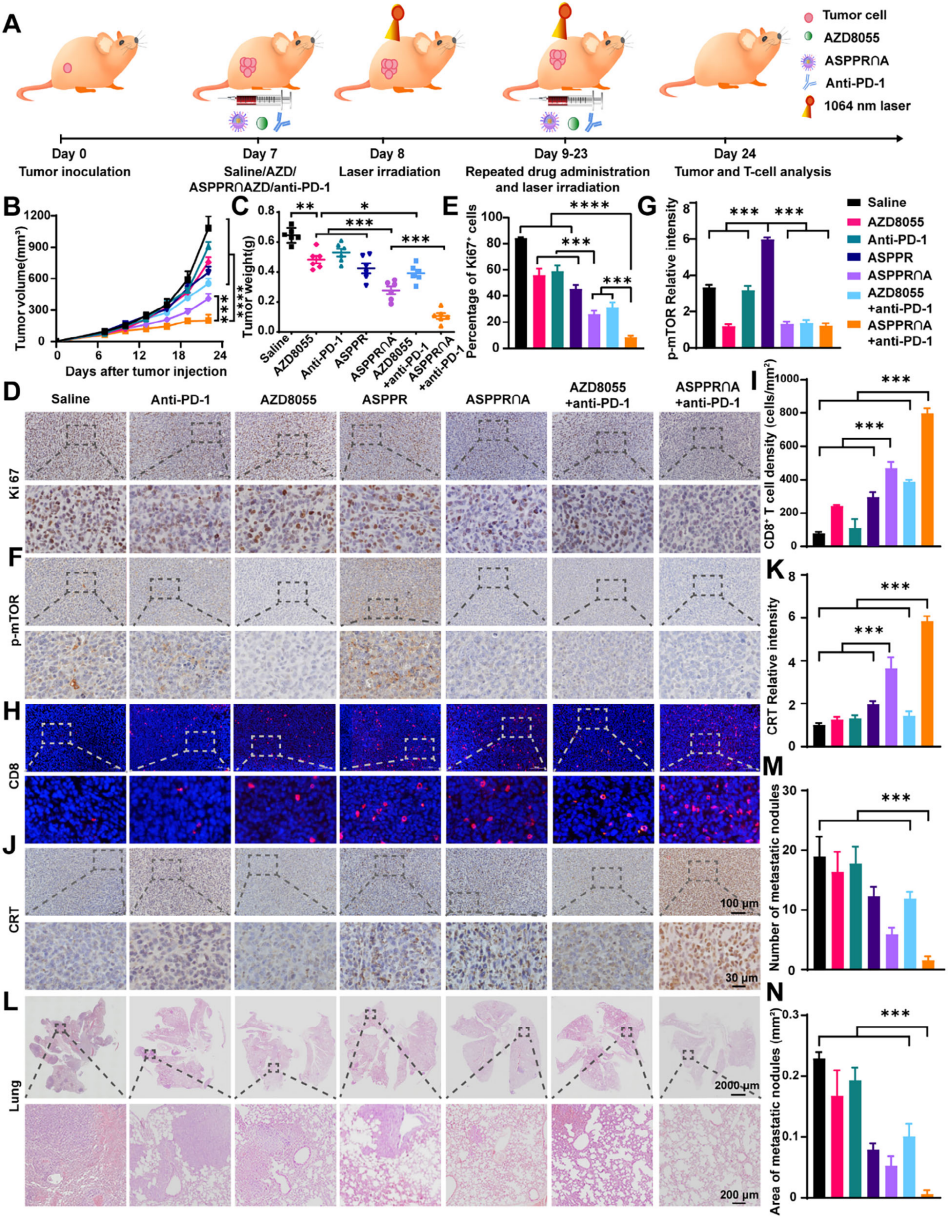
ASPPR∩A‐mediated PTT and targeted‐therapy synergistically inhibit tumor growth and enhance TILs. A) Schematic illustration of ASPPR∩A‐based thermo‐targeted and anti‐PD‐1 combination therapy. B,C) Orthotopic 4T1‐bearing mice were *i.v*. injected with saline (control), free AZD8055, ASPRP vehicle with laser, or ASPPR∩A with laser. The average tumor growth curves were profiled (B), and tumors were weighed after animal euthanasia (C, day 24). The results are presented as mean ± SD (n = 6). D) Proliferative cells were visualized by Ki‐67 IHC. Scale bar 100 µm. E) The relative intensity of Ki‐67 in each group was quantified. F) The activation of the mTOR pathway following PTT was visualized through p‐mTOR. IHC staining. Scale bar 100 µm. G) The relative intensity of p‐mTOR in each group was quantified. H) CD8^+^ T‐cells were visualized by CD8 immunofluorescence staining. Scale bar 100 µm. I) The relative intensity of CD8^+^ T cells in each group was quantified. J) The expression of CRT was visualized by IHC staining, scale bar 100 µm. K) The relative intensity of CRT in each group was quantified. L) Hematoxylin and eosin (H&E) staining of the whole lung tissue. M,N). Number (M) and area (N) of pulmonary metastases in tumor‐bearing mice. Data are shown as mean ± SD (n = 3). Statistical analysis was measured by one‐way ANOVA, ^*^ *p* < 0.05, ^**^ *p* < 0.01, and ^***^ *p* < 0.001.

Encouraged by the promising performance of the ASPPR nanosystem in tumor‐specific cellular uptake, photothermal effects, in vitro oncolytic and immune regulation functions, we further evaluated the synergistic efficacy of ASPPR‐encapsulated AZD8055 combined with immunotherapy in vivo. BALB/c mice bearing 4T1 tumors were randomly divided into seven groups: saline, anti‐PD‐1, AZD8055, ASPPR empty vector, ASPPR∩A, anti‐PD‐1+AZD8055, and anti‐PD‐1+ASPPR∩A. The treatment regimen is outlined in **Figure** **5**A. Briefly, AZD8055, ASPPR empty vector, and ASPPR∩A were administered intravenously every 3 days via tail vein injection. In the ASPPR empty vector, ASPPR∩A, and anti‐PD‐1+ASPPR∩A groups, the tumors were irradiated with a 1064 nm laser (0.5 W cm^−2^) for 5 min, 24 h after ASPPR administration, a timepoint selected based on in vivo imaging results showing peak tumor accumulation of ASPPR nanocomposites (Figure 3E). Anti‐PD‐1 antibody was administered every 6 days. Tumor growth curves were meticulously plotted during the treatment period, and tumor weights were recorded at the end of the treatment (Figure 5B,C; Figure s6, Supporting Information). Consistent with previous studies,^[^ ^11^ ^]^ the anti‐PD‐1 monotherapy showed limited tumor suppression in 4T1 breast cancer. The free AZD8055 exhbited only modest antitumor activity, showing minimal synergistic effects when co‐administered with the anti‐PD‐1 antibody. Interestingly, the laser‐irradiated ASPPR empty vector also showed a certain degree of tumor‐suppressive capacity, indicating the potential antitumor activities of ASPPR‐mediated PTT. In comparison to treatment with either AZD8055 or the ASPPR empty vector alone, the application of ASPPR∩A resulted in marked tumor growth inhibition and significantly enhanced the antitumor efficacy of the anti‐PD‐1 antibody, indicating a synergistic interaction between ASPPR∩A and PD‐1 blockade. The combination of ASPPR∩A with anti‐PD‐1 treatment yielded the most pronounced tumor growth inhibition among all treatment groups. The status of tumor cell proliferation was assessed through Ki67 immunohistochemical (IHC) staining and subsequent quantification (Figure 5D,E). Compared to the free AZD8055 and laser‐irradiated empty ASPPR nanocomposites, ASPPR∩A with laser irradiation significantly decreased the number of Ki67^+^ cells within the tumor tissues. This finding suggests a synergistic effect between PTT and mTOR‐targeted therapies, which was further enhanced by anti‐PD‐1 treatment.

We then sought to understand the mechanism by which ASPPR∩A nanosystem synergizes with anti‐PD‐1 treatment. IHC analysis demonstrated that ASPPR‐mediated PTT significantly upregulated phosphorylation of AKT, mTOR, S6, and 4EBP1, indicating thermal stress‐induced PI3K/AKT/mTOR hyperactivation (Figure 5F,G; Figure S7A–F, Supporting Information). AZD8055 suppressed this phosphorylation cascade and reversed pathway overactivation, confirming that mTOR inhibition disrupts tumor adaptive compensation to photothermal therapy, enhancing therapeutic sensitivity. CD8^+^ T‐cells in the tumor microenvironment were visualized by immunofluorescence, and cell densities were quantified. The results showed that ASPPR∩A with laser irradiation significantly increased the number of CD8^+^ T‐cells compared with free AZD8055 and ASPPR vehicle with laser irradiation alone (Figure 5H,I), suggesting a synergistic effect between mTOR‐targeted therapy and PTT. This was mainly attributed to the nanoplatform design of ASPPR∩A, in which the EPR effect combined with pH‐responsive release enabled higher tumor accumulation and intracellular delivery of AZD8055, leading to more effective inhibition of mTOR signaling and alleviation of immunosuppression. In parallel, the photothermal effect of the gold nanocage promoted the release of tumor‐associated antigens and DAMPs, thereby facilitating the infiltration of CD8⁺ T cells. Importantly, the combination of ASPPR∩A and anti‐PD‐1 treatment significantly increased the number of CD8^+^ T‐cells. The phenomenon was further validated by multiparameter flow cytometry analysis (Figure S8A–G, Supporting Information). Consistent with the immunohistochemistry results, ASPPR∩A‐mediated thermo‐targeted therapy synergistically increased the abundance of CD3⁺ lymphocytes and cytotoxic T lymphocytes (CTLs, CD3⁺CD8⁺), and T helper cells (CD3⁺CD4⁺) within tumors. This coordinated T‐cell infiltration was further augmented by PD‐1 blockade. Moreover, the treatment significantly enhanced the infiltration of total dendritic cells (CD11c⁺) and promoted their maturation (CD11c^+^CD86^+^). These comprehensive results suggest that the thermo‐targeted‐immune regimen effectively converts immune “cold” tumors into “hot” ones. Interestingly, we found the combinatory treatment significant upregulate the expression of PD‐L1 on tumor cells (Figure S8H,I, Supporting Information). This is likely an adaptive response driven by IFN‐γ released from the robustly infiltrated T cells, a well‐characterized feedback mechanism.^[^ ^39^ ^]^ Importantly, this induced PD‐L1 expression enhances the target availability for the anti‐PD‐1 antibody, thereby reinforcing the efficacy of the checkpoint blockade and contributing to the overall therapeutic synergy.

Further mechanism dissection demonstrated that ASPPR∩A enhances T‐cell infiltration by intracellular hyperthermia and oncogene inhibition, synergistically inducing ICD in tumor cells and releasing tumor‐associated neoantigens. The ICD marker proteins, including CRT, HMGB1, HSP27, and HSP70, were then detected by IHC. We observed a significant increase in ICD levels in photothermally treated tumors (ASPPR) compared to controls (Figure 5J,K; Figure S9A–F, Supporting Information). More importantly, the ICD levels in laser‐irradiated ASPPR∩A were significantly higher than those in tumors treated with AZD8055 or the ASPPR vector alone, suggesting a synergistic effect between hyperthermia and mTOR‐targeted therapy. To confirm the observed ICD, tissue necrosis was examined via H&E staining (Figure S9G, Supporting Information). Consistently, tumors treated with the combined regimen (ASPPR∩A) exhibited more extensive necrosis than those treated with AZD8055 or the ASPPR vector. Thus, the ASPPR∩A induced ICD and tumor‐associated neoantigens promote T cell infiltration, which enhances the antitumor efficiency of PD‐1 blockade.

Since 4T1 breast cancer easily spontaneous metastasizes to the lung, and ASPPR∩A can boost antitumor immune response, we wonder whether this combination treatment regimen could inhibit tumor metastasis. To validate this speculation, we detected the micrometastatic lesion in the lung tissue via H&E staining. Consistent with the tumor growth curve (Figure 5L–N), anti‐PD‐1 single treatment did not inhibit tumor metastasis. In addition, AZD8055 combined with anti‐PD‐1 treatment slightly inhibited tumor metastasis. Excitingly, the ASPPR∩A nanosystem‐mediated photothermal and mTOR‐targeted therapy significantly inhibited lung metastasis, with anti‐PD‐1 treatment further enhancing their anti‐metastasis effect (Figure 5L–N). We also evaluated the preliminary safety profile of the ASPPR∩A nanocomposites. First, we assessed potential T‐cell toxicity induced by PTT via co‐staining of CD8 and TUNEL apoptosis markers in treated tumor tissues (Figure S10A, Supporting Information). While varying degrees of apoptosis were observed across treatment groups, particularly in the ASPPR∩A group, minimal co‐localization of apoptotic signals with CD8⁺ T cells was detected, indicating low cytotoxicity toward cytotoxic T lymphocytes. We then assessed their systematic toxicity, and found the mice were well‐tolerated to the nanocomposites. No deaths or significant side effects were observed in any group. Hemolysis assays confirmed that ASPPR nanocomposites demonstrated good hemocompatibility (Figure S10B,C, Supporting Information). Histopathological examination of major organs, including the heart, liver, spleen, lungs, and kidneys, showed no evidence of pathological damage or necrosis (Figure S10D, Supporting Information). Considering that the lung is another major organ that nanoparticle accumulation (Figure 3E), we further evaluated the degree of pulmonary fibrosis using Masson's trichrome staining, a gold‐standard method for assessing lung damage and fibrosis. The results demonstrated that all drug‐treated mice maintained normal lung architecture with typical collagen distribution (Figure S10E, Supporting Information). Furthermore, serum biochemical analysis of liver enzymes (ALT, AST), lactate dehydrogenase (LDH), renal function markers, and cardiac enzymes showed no significant abnormalities (Figure S10F–J, Supporting Information), collectively supporting the biosafety of the ASPPR nano‐system for in vivo application.

Taken together, these results demonstrated that the combined regimen of PTT and mTOR‐targeted therapy effectively activates the antitumor immune response by enhancing neoantigen release, significantly boosting the efficacy of PD‐1 immunotherapy in immune “cold” tumors. This thermo‐targeted‐immune combinatorial regimen not only inhibits primary tumor growth but also has the potential to destroy the micrometastatic lesion.

### 2.6 Local Administration of ASPPR‐mTORi Fueled the Systematic ICB Therapy

**Figure 6:**
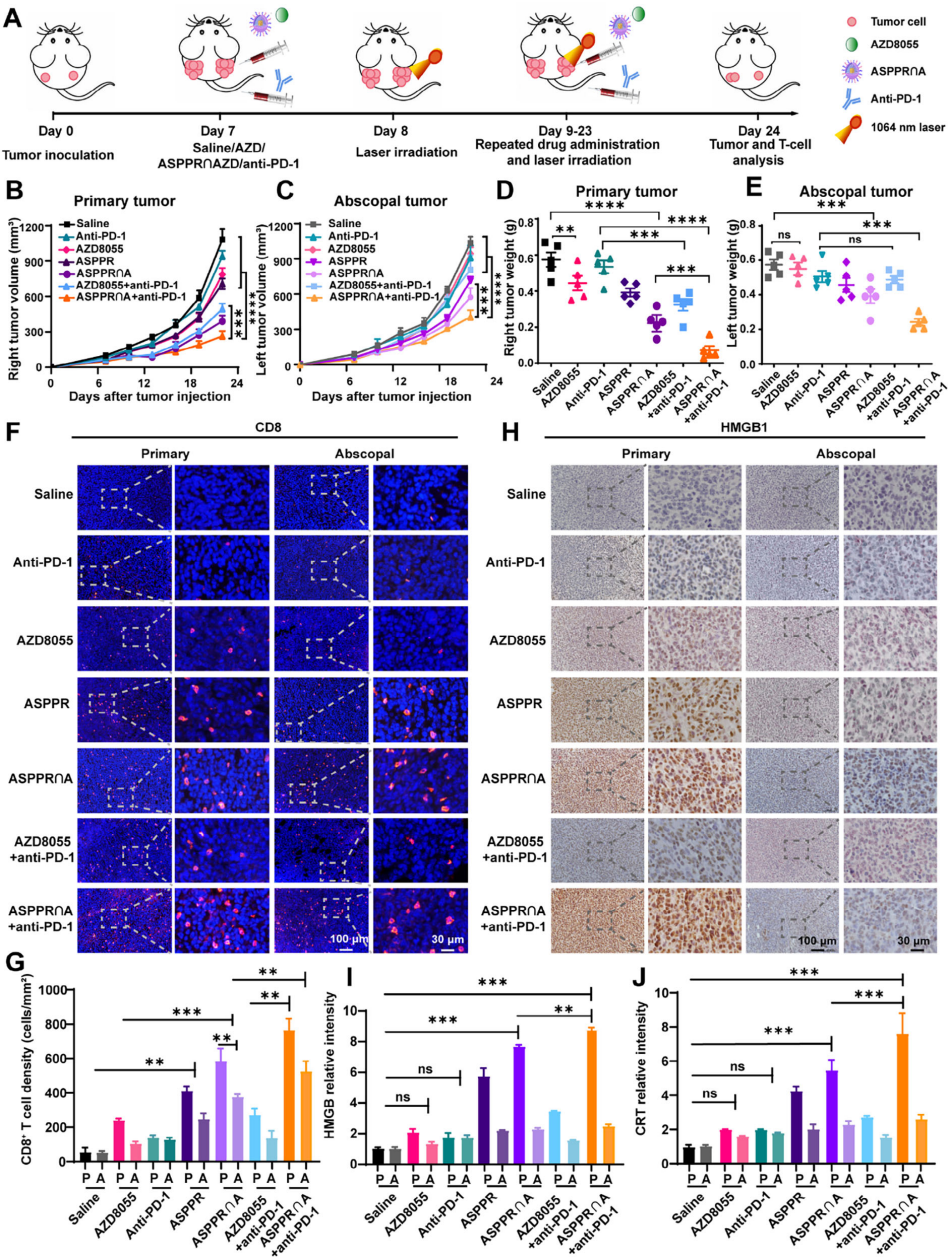
Synergistic inhibition of primary and abscopal tumor growth by ASPPR∩A and anti‐PD‐1 combination therapy. A) Schematic illustration of ASPPR‐AZD8055‐based thermo‐targeted and anti‐PD‐1 combination therapy to inhibit primary tumor (right) and abscopal tumor (left). The right tumors were intratumorally (*i.t*.) injected with saline, AZD8055, or ASPPR∩AZD8055 nanocomposite, the left tumors were untreated abscopal tumors. Anti‐PD‐1 antibodies were *i.v*. injected. B,C) The average tumor growth curves of primary tumor (B) and abscopal tumor (C) in mice treated with saline (control), anti‐PD‐1, free AZD8055, ASPPR with laser, ASPPR∩A with laser, and ASPPR∩A with laser plus anti‐PD‐1, respectively. D,E) The average tumor weight of primary tumor (D) and abscopal tumor (E) in mice treated with saline (control), anti‐PD‐1, free AZD8055, ASPPR with laser, ASPPR∩A with laser, ASPPR∩A with laser plus anti‐PD‐1, respectively. The results are presented as mean ± SD (n = 5). F) CD8^+^ T‐cells were visualized by immunofluorescence staining. Scale bar 100 µm. G) The relative intensity of CD8^+^ in each group was quantified. H) ICD was visualized by HMGB1 IHC staining. Scale bar 100 µm. I) The relative intensity of HMGB1 in each group was quantified. J) The relative intensity of CRT in each group was quantified. P, primary tumor. A, abscopal tumor. Statistical analysis was measured by one‐way ANOVA (n=3). ns, not significant, ^*^ *p* < 0.05, ^**^ *p* < 0.01, and ^***^ *p* < 0.001.

In the 4T1 breast cancer spontaneous metastasis model, we observed an encouraging phenomenon that ASPPR∩A and anti‐PD‐1 combinatorial treatment effectively eradicated lung micrometastatic lesions (Figure 5L). These findings underscore the dual functions of thermo‐targeted therapy and immunotherapy in managing primary tumors and unresectable metastatic sites by enhancing systemic antitumor immune responses. Therefore, we further investigated the therapeutic potential of this combination approach in addressing unresectable metastatic lesions within multifocal tumor scenarios. To this end, we established a bilateral tumor model by inoculating 4T1 breast cancer cells on the right and left flanks of mice. ASPPR∩A‐mediated PTT combined with mTOR targeted therapy was locally applied to the tumor on the right flank, and the anti‐PD‐1 antibody was administered systemically via tail vein injection (**Figure** **6**A). Tumor volumes and growth curves for both primary and distant tumors were recorded every three days, respectively (Figure 6B,C; Figure S11, Supporting Information). At the end of the treatment (day 24), tumors were dissected and weighed (Figure 6D,E). The results showed that intratumoral injection of AZD8055 inhibited primary tumor growth. However, it did not exert an impact on the non‐treated distant tumor. Similarly, the combination treatment of AZD8055 with anti‐PD‐1 yielded analogous results. On the contrary, the localized administration of ASPPR∩A plus laser significantly suppressed the growth of both primary and abscopal tumors, exhibiting a synergistic effect when combined with PD‐1 blockade (ASPPR∩A + anti‐PD‐1). Notably, significant growth inhibition was also observed in abscopal tumors of mice treated with either ASPPR∩A alone or in combination with anti‐PD‐1 antibody, suggesting that the local application of PTT‐mTOR targeted therapy resulted in a systemic therapeutic benefit.

In the above results, we observed that ASPPR∩A‐mediated PTT and mTOR‐targeted therapy could induce the ICD of tumors, which stimulates antitumor immune response and augments the infiltration of tumor‐specific CD8^+^ T cells (Figure 5F). Thus, we speculate that this synergistic approach leads to the suppression of distant, untreated tumors through the enhancement of T cell presence within their respective tumor microenvironments. To validate this hypothesis, we visualized CD8^+^ T cells in both the treated primary tumor and the untreated distant tumor (Figure 6F). The results demonstrated that, compared with AZD8055 monotherapy, the combination of PTT with mTOR‐targeted therapy (ASPPR∩A plus laser) significantly promoted CD8^+^ T cell infiltration in both the primary and distant tumors (Figure 6F, line 5). Further quantitative analysis revealed that the density of CD8^+^ T cells in the distant tumor was slightly lower than in the primary tumor (Figure 6F), suggesting that effective systemic antitumor immune responses were achieved through primary tumor oncolysis. On top of the ASPPR∩A treatment, systemic administration of the anti‐PD‐1 antibody further enhanced CD8^+^ T‐cell infiltration (ASPPR∩A plus anti‐PD‐1 group, Figure 6F, line 7). The analysis of ICD markers corroborated our previous findings; we assessed the expression levels of key ICD indicators, including HMGB1, CRT, HSP70, and HSP27, in both primary and distant tumors. PD‐1 blockade and AZD8055 treatment either alone or in combination did not increase the expression of these ICD markers in both primary and distant tumors (Figure 6H; Figure S12A–C, Supporting Information, line 2, 3). Whereas, ASPPR∩A with laser irradiation significantly induced the expression of these ICD markers in the primary tumor (Figure 6H; Figure S12A–C, Supporting Information, line 4), and this difference was further enhanced by the addition of the anti‐PD‐1 antibody (Figure 6H; Figure S12A–C, Supporting Information, line 7). Intriguingly, the expression levels of ICD markers in distant tumors remained unchanged following treatment with ASPPR∩A plus laser. These results underscore the role of localized PTT and mTOR‐targeted therapy in inducing ICD and the release of neoantigens, ultimately facilitating the systemic efficacy of immune checkpoint blockade therapies.

## 3 Discussion

**Figure 7:**
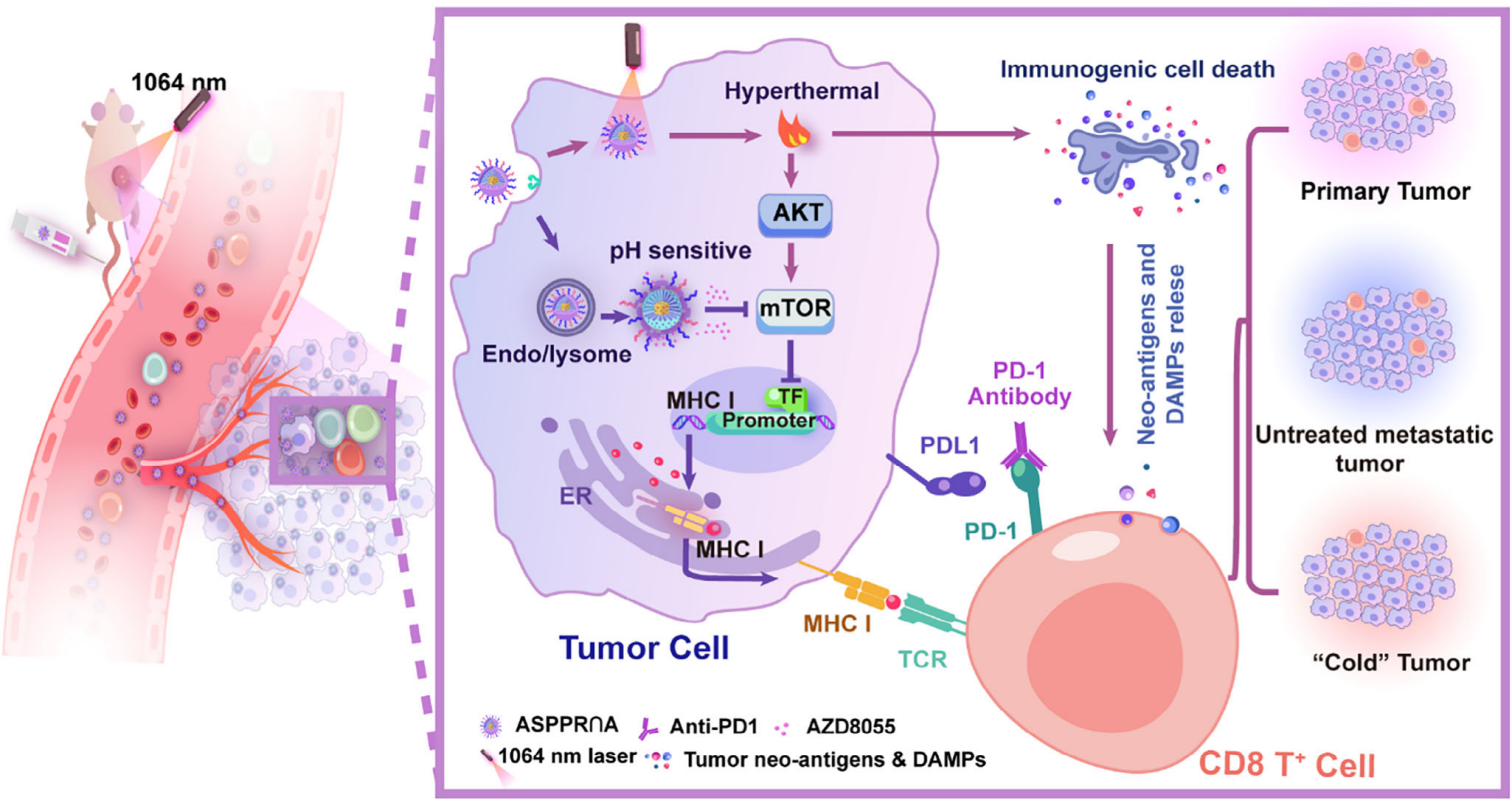
Schematic of the ASPPR∩A nanocomposite‐enabled thermally‐targeted immune strategy for tumor treatment. The *i.v*. injected ASPPR∩A nanocomposite is specifically enriched in the tumor microenvironment and internalized by tumor cells. In the acidic endosomes/lysosomes of tumor cells, the drugs were released from the nanocomposite to inhibit the AKT‐mTOR pathway and enhance MHC‐I antigen presentation. Concurrently, upon exposure to laser irradiation, the gold nanocomponents convert light energy into thermal energy, creating a synergistic effect with the mTOR inhibitors to induce cytotoxicity in tumor cells and promote ICD. This dual‐action strategy, comprising PTT and mTOR inhibition, not only synergistically triggers tumor ICD and enhances the release of neoantigens but also counters PTT‐induced mTOR oncogene activation, ultimately promoting MHC‐I antigen presentation. The resultant increased neoantigen release and improved antigen presentation stimulate a systemic antitumor immune response characterized by enhanced T cell infiltration. Subsequently, tumor‐specific T cells are activated by the anti‐PD‐1 antibodies, thereby facilitating effective therapeutic outcomes against primary tumors, untreated metastatic lesions, and immune “cold” tumors.

PTT represents a significant advancement in overcoming the limitations of ICB treatment for TNBC. Previous studies have established that PTT enhances antitumor immunity by inducing ICD and releasing tumor‐associated antigens to activate antigen‐presenting cells (APCs) like DCs,^[^ ^16^, ^40^ ^]^ initiating a T‐cell‐mediated antitumor immune response. However, the adaptive activation of pro‐survival pathways, such as AKT/mTOR, has been largely overlooked. For instance, research has indicated that hyperthermia triggers AKT/mTOR signaling to promote normal cell growth.^[^ ^20^, ^21^ ^]^ Our results demonstrated that while PTT enhances ICD of TNBC cells, the AKT/mTOR pathway was highly activated (Figure 1A–C). Consequently, combining PTT with mTOR‐targeted therapy is viewed as a promising strategy to improve the effectiveness of ICB therapy in TNBC. Unfortunately, clinical trials of mTOR inhibitors (e.g., everolimus) in TNBC have shown limited efficacy due to poor tumor targeting and immunosuppressive side effects.^[^ ^41^, ^42^ ^]^ Based on these insights, our study introduces a dual‐modality approach that not only addresses the PTT‐mTOR paradox but also leverages nanotechnology to achieve spatiotemporal control of therapy. We developed a pH/NIR‐II‐responsive ASPPR∩A nanosystem to synergize localized hyperthermia with mTOR inhibition. This combination promotes MHC‐I antigen presentation in tumor cells and ICD‐mediated antigen presentation in DCs simultaneously. The combined PTT and mTOR‐targeted therapy significantly enhanced the effectiveness of PD‐1 blockade and suppressed tumor metastasis (**Figure** **7**). This strategy resolves a critical gap in prior PTT and ICB combinations by mechanistically interrupting tumor adaptation pathways, offering a blueprint for converting immunologically “cold” tumors into “hot” ones.

The ASPPR∩A nanosystem represents a breakthrough in overcoming the limitations of conventional therapies by integrating NIR‐II photothermal precision, pH‐responsive drug release, and tumor‐targeted delivery. While gold‐based PTT agents (e.g., Au nanorods) are well‐documented for their photothermal efficiency, most operate in the NIR‐I window (700–900 nm) with limited tissue penetration.^[^ ^33^, ^43^ ^]^ Inspired by recent advances in NIR‐II phototherapy,^[^ ^44^ ^]^ we engineered a gold nanocage (AuNC) with a surface plasmon resonance peak at 1064 nm, achieving deeper penetration and higher photothermal conversion efficiency than traditional NIR‐I systems.^[^ ^45^ ^]^ Compared with other NIR‐II nanomaterials, such as semiconducting polymers, rare‐earth nanocrystals, and organic dyes, Au nanocages offer superior photothermal conversion efficiency, excellent photostability, facile surface functionalization, and well‐documented biocompatibility, making them a highly attractive and translationally relevant platform for 1064 nm‐mediated photothermal therapy.^[^ ^16^, ^46^, ^47^, ^48^, ^49^, ^50^ ^]^ Furthermore, unlike pH‐insensitive carriers^[^ ^51^ ^]^ or conventional mTOR inhibitor delivery platforms (e.g., liposomes),^[^ ^52^ ^]^ which lack spatiotemporal control, our system features a “core‐shell‐membrane” architecture: an NIR‐II‐responsive AuNC core, a mesoporous silica shell encapsulating mTOR inhibitor, and a pH‐sensitive PAA layer conjugated with the iRGD tumor‐homing peptide. This design enables tumor‐specific drug release through PAA protonation in the acidic tumor microenvironment^[^ ^35^, ^53^, ^54^ ^]^ while the iRGD peptide enhances tumor accumulation ability.^[^ ^55^ ^]^ Crucially, the spatiotemporal synergy between NIR‐II‐triggered hyperthermia induces ICD‐mediated immune activation, and mTOR pathway inhibition reverses MHC‐I suppression, a dual mechanism absent in earlier PTT or mTOR monotherapy approaches. Furthermore, ASPPR∩A maintains stable photothermal performance (ΔT > 35 °C after laser irradiations). Additionally, ASPPR∩A maintains stable photothermal performance after multiple laser irradiations. This multifunctional integration not only overcomes the trade‐offs between photothermal efficacy and drug delivery precision but also establishes a new paradigm for synergistic thermo‐immunotherapy in aggressive cancers.

Mechanistically, our study bridges a knowledge gap in AKT‐mTOR‐driven immune evasion. Earlier reports linked AKT‐mTOR hyperactivity to MHC‐I downregulation in mouse melanoma cells, but clinical evidence remained sparse.^[^ ^56^ ^]^ By analyzing TNBC patient samples, we demonstrated a robust inverse correlation between mTOR activity and MHC‐I expression, confirming the clinical relevance of this immunosuppressive axis. MHC‐I antigen presentation is essential for immune surveillance, as it enables CD8⁺ T cells to recognize and eliminate tumor cells by presenting intracellular antigens.^[^ ^57^, ^58^ ^]^ AKT/mTOR hyperactivation in TNBC suppresses the key transcription factor IRF1, leading to attenuated expression of both MHC‐I heavy chains and critical components of the antigen processing machinery.^[^ ^56^, ^59^ ^]^ This coordinated downregulation impairs tumor antigen presentation, rendering tumor cells essentially “invisible” to CD8⁺ T cells and thereby facilitating immune evasion and tumor progression. Our therapeutic intervention addresses this limitation through localized mTOR inhibition via the ASPPR∩A nanosystem, which restores MHC‐I expression and re‐establishes tumor cell visibility to the immune system. Furthermore, the nanocomposite‐mediated PTT induces robust ICD to enhance tumor antigen release and dendritic cell activation. This dual mechanism creates a self‐reinforcing cycle of immune activation: newly released antigens can be effectively processed and presented by both dendritic cells to prime naive T cells and by tumor cells themselves to enable recognition by activated CD8⁺ T cells. This mechanistic synergy represents an effective strategy for converting immunologically “cold” tumors into “hot” ones susceptible to immune‐mediated destruction. Our findings redefine AKT‐mTOR signaling as a master regulator of both tumor cell survival and immune escape that can be therapeutically targeted to enhance checkpoint blockade efficacy.

Although we meticulously optimized the ASPPR∩A nanosystem for tumor‐specific delivery and synergistic efficacy, several limitations warrant consideration. First, while PTT‐induced AKT/mTOR activation was consistently observed, the precise molecular mechanism remains unclear. This phenomenon may involve stress‐responsive pathways, such as heat shock protein signaling or ROS‐mediated AKT activation, which require further investigation. Second, despite ASPPR∩A showing minimal lymphocyte uptake, its impact on myeloid‐derived suppressor cells (MDSCs) and tumor‐associated macrophages (TAMs), key players in tumor immunosuppression,^[^ ^60^ ^]^ remains uncharacterized. The off‐target needs to be further validated. Third, while acute toxicity was absent in murine models, long‐term safety, particularly cumulative photothermal damage or gold nanoparticle accumulation in vital organs, remains uncharacterized and demands rigorous assessment in large‐animal studies before clinical translation. Taken together, our study establishes a proof‐of‐concept strategy for harmonizing PTT‐driven immunogenicity with mTOR pathway blockade to potentiate PD‐1 blockade in TNBC. Future studies should focus on elucidating the PTT‐mTOR crosstalk mechanism, engineering immune cell‐exclusive targeting modalities, and validating long‐term biosafety. This approach may extend beyond TNBC to other immunologically “cold” malignancies and metastatic tumors, offering a promising strategy for precision thermo‐immunotherapies in oncology.

## 4 Experimental Section

### Materials

Chloroauric acid trihydrate (HAuCl_4_·3H_2_O, 99%), tetraethoxysilane (TEOS, 98%), polyvinylpyrrolidone (PVP, molecular weight ≈55000), polyacrylic acid (PAA) and 3‐(methacryloyloxy)propyltriethoxysilane (MPS) were all purchased from Sigma–Aldrich, sodium chloride (NaCl), Silver nitrate (AgNO_3_, 99.8%) and ethylene glycol (EG) were obtained from Beijing Chemical (Beijing, China). Nile Red and AZD8055 were purchased from Med Chem Express. All chemicals were utilized as received without additional purification. The anti‐PD‐1 antibody (RRID: AB_3028506) was generously provided by Chengdu Kangmei Biotechnology Co., Ltd. (Chengdu, China). Penicillin, trypsin, and streptomycin were sourced from Merck Millipore (USA), while fetal bovine serum (FBS) was obtained from Gibco Life Technologies. RPMI and DMEM media were purchased from Hyclone (USA).

### Cells and Mice

Triple‐negative breast cancer (TNBC) cell lines 4T1(RRID: CVCL_0125), MDA‐MB‐468 (RRID: CVCL_0419), and BT549 (RRID: CVCL_1092) were obtained from the American Type Culture Collection (ATCC). The EL4 (RRID: CVCL_0255) and Jurkat (RRID: CVCL_0065) lymphocyte cell lines were also sourced from ATCC. PBMCs were isolated from healthy donor blood samples (RRID: CVCL_9T50). These cell lines were cultured in DMEM or RPMI medium supplemented with 10% FBS, 100 U mL^−1^ penicillin, and 100 µg mL^−1^ streptomycin. Cells were incubated at 37 °C with 5% CO_2_. All cell lines were routinely tested and confirmed to be free of mycoplasma contamination. These cell lines were selected as representative models for TNBC and immune response studies due to their well‐established characteristics in tumor growth, immune interactions, and therapeutic response profiling. Their use supports the validity of the conclusions regarding the therapeutic efficacy and immunomodulatory mechanisms investigated in this work. Female BALB/c mice were obtained from Beijing HFK Bioscience Co., Ltd. (Beijing, China) and housed under specific pathogen‐free (SPF) conditions. Humane care was provided throughout the study, and all animal experiments followed protocols approved by the Animal Ethics Committee of Sichuan University. The procedures complied with the NIH Guide for the Care and Use of Laboratory Animals and the Animal Welfare Act.

### Gold Nanocage (AuNC) Synthesis

AuNCs were prepared via a galvanic replacement reaction involving silver nanocubes (AgNCs) and chloroauric acid (HAuCl_4_). To synthesize AgNCs, 35 mL of ethylene glycol (EG) was preheated at 160 °C for 1 h, followed by adding 0.25 g polyvinylpyrrolidone (PVP) dissolved in 12 mL EG. Next, 0.45 mL of a 3 mM sodium sulfide solution in EG was introduced, and 2.5 mL of a 282 mM silver nitrate (AgNO_3_) solution in EG was gradually added, forming 60–70 nm AgNCs. The resulting nanocubes were purified by centrifugation with ethanol and deionized water. For AuNC synthesis, 500 µL of the purified AgNCs (3 nM) was combined with 5 mL deionized water containing PVP (1 mg mL^−1^) and heated at 100 °C for 10 min. A slow addition of 0.5 mM HAuCl4 solution continued until UV spectrophotometry confirmed the optical absorption peak. The mixture was refluxed for another 30 min and cooled to room temperature. Centrifugation removed AgCl using a saturated sodium chloride solution, followed by repeated water washes to eliminate residual PVP and NaCl.

### Preparation of Mesoporous Silica‐Coated AuNCs (AuNC@mSiO2)

A mesoporous silica layer was synthesized using a sol‐gel process. Initially, 3 mL of AuNCs were mixed with 13.8 mL of isopropanol. While stirring magnetically, 0.4 mL of aqueous ammonia (28 wt%) and 0.04 mL of tetraethoxysilane (TEOS) were added sequentially. The mixture was stirred at room temperature for 16 h, forming AuNCs with a 20 nm silica shell (AuNC@SiO_2_). The product was purified via centrifugation at 8000 g for 20 min using ethanol. To transform the solid silica shell into a porous structure, a surfactant‐assisted selective etching method was applied. Specifically, 100 mg of AuNC@SiO_2_ particles were dispersed in 15 mL of water, sonicated, and treated with 200 mg of sodium hydroxide (NaOH) under magnetic stirring. The reaction was maintained at 50 °C for 10 h. The final product, AuNC@mSiO_2_ nanoparticles with a 20 nm mesoporous silica shell, was collected by centrifugation and thoroughly washed with deionized water and ethanol.

### Preparation of AuNC@mSiO2@Copolymer Nanocomposites

10 mL of AuNC@mSiO_2_ was dispersed in 20 mL of isopropanol, followed by the slow addition of 50 µL of 3‐aminopropyltriethoxysilane (APTES). The solution was stirred at 50 °C for 24 h. After cooling to room temperature, the solution was centrifuged, and the precipitate was washed alternately with ethanol and water. The resulting product was dried in a vacuum oven at 60 °C for 12 h, yielding AuNC@mSiO_2_‐NH_2_ nanoparticles.

2 mL of a 10 mg mL^−1^ methanol suspension of AuNC@mSiO_2_‐NH_2_ nanoparticles was mixed with 200 µL of a 10 mg mL^−1^ methanol solution of either Nile Red or AZD8055. The mixture was incubated in the dark for 12 h. After incubation, the particles were collected by centrifugation at 10,000 rpm for 15 min, washed three times with phosphate‐buffered saline (PBS), and then lyophilized. The drug loading (LC) was calculated using the following formula:

(1) $$\begin{array}{ccc} \mathrm{LC} & = & \left(\mathrm{Total}\;\mathrm{amount}\;\mathrm{of}\;\mathrm{drug}-\mathrm{Amount}\;\mathrm{of}\;\mathrm{drug}\;\mathrm{in}\;\mathrm{the}\;\mathrm{supernatant}\right)\\ & & /\mathrm{Total}\;\mathrm{amount}\;\mathrm{of}\;\mathrm{drug}\times 100\% \end{array}$$

100 mg of Nile Red‐ or AZD8055‐loaded AuNC@mSiO_2_‐NH_2_ nanoparticles were dispersed in 40 mL of deionized water. The resulting solution was then slowly added dropwise into an aqueous solution of polyacrylic acid (PAA, molecular weight = 1800, concentration = 5 mg mL^−1^) while undergoing ultrasonic treatment. After stirring for 2 h, the mixture was centrifuged at 12,000 rpm for 5 min. The pellet was redispersed in deionized water for further use, yielding PAA‐coated dye‐ or drug‐loaded AuNC@mSiO_2_‐NH_2_ nanoparticles (AuNC@mSiO_2_@PAA).

50 mg of mPEG‐5K‐NH_2_ was added to 200 mL of a 5 mg mL^−1^ aqueous suspension of AuNC@mSiO_2_‐PAA (pH 7.5), and the mixture was sonicated for 30 min. After stirring overnight, the mixture was purified using a 100 kDa molecular weight cutoff ultrafiltration device, followed by three washes with deionized water.

The resulting AuNC@mSiO_2_@PAA@PEG‐iRGD nanoparticles were redispersed in water and stored at 4 °C, labeled as ASPPR. To investigate the pH‐dependent release characteristics of AZD8055 from ASPPR nanoparticles, equal amounts of four nanoparticle samples were immersed in 1 mL of PBS solutions with pH values of 7.4, 6.8, 5.5, and 4.5. At various time points, the samples were centrifuged, and the supernatants were collected. The absorbance values of the supernatants were measured using a microplate reader, and the concentration of released AZD8055 was calculated based on these measurements.

### Sample Characterization

The optical characteristics of the materials were assessed using a Bio‐Tek microplate reader (SYNERGY H1) and a Hitachi U‐4100 UV/Vis/NIR spectrophotometer. Transmission electron microscopy (TEM) images were obtained on a JEOL JEM‐2100Plus operating at 80.0 kV. Nitrogen adsorption–desorption isotherms were analyzed using a Micromeritics ASAP 2020 M automated analyzer. Particle size and Zeta potential of the nanoparticles were determined using a PARTICLE METRIX ZETAVIEW nanoparticle tracking analyzer. The measurement conditions and parameters for DLS and zeta potential measurements are shown in Table S3 (Supporting Information). Fourier‐transform infrared (FTIR) spectra were recorded with a Bruker Vertex 70 FT‐IR spectrometer.

### Isolation of Human PBMCs

PBMCs were isolated from healthy donor blood samples using density gradient centrifugation. Briefly, whole blood was diluted 1:1 with PBS and carefully layered over human peripheral blood lymphocyte separation medium (Beyotime Biotechnology, #C0025) in a 50 mL centrifuge tube. Samples were centrifuged at 400×g for 30 min at room temperature with the brake turned off. After centrifugation, the PBMC layer was carefully collected using a sterile pipette and transferred to a new tube. The cells were washed twice with PBS by centrifugation at 300×g for 10 min to remove platelets and residual peripheral blood lymphocyte separation medium. The resulting PBMCs were resuspended in RPMI‐1640 medium supplemented with 10% FBS, 100 U mL^−1^ penicillin, and 100 µg mL^−1^ streptomycin, and cultured at 37 °C in a humidified atmosphere containing 5% CO_2_.

### Cellular Uptake and Biodistribution of ASPPR Nanocomposites

To assess the uptake of ASPPR∩A nanocomposites, various cell types, including 4T1, BT549, MDA‐MB‐468, EL4, Jurkat cells, and human peripheral blood mononuclear cells (PBMC), were used. Briefly, 5 × 10⁴ cells were plated in 12‐well plates and allowed to grow overnight. The cells were then exposed to varying concentrations of ASPPR∩A nanocomposites for 24 h. After treatment, the cells were harvested and analyzed via flow cytometry. For time‐dependent uptake analysis, cells were treated with 50 µg mL^−1^ ASPPR∩A nanocomposites and analyzed at 1, 4, 8, and 24‐h using flow cytometry.

The cellular localization of ASPPR nanocomposites was investigated via confocal microscopy. Tumor cells and lymphocytes were cultured on cover slips in 12‐well plates. After 24 h of incubation with 50 µg mL^−1^ ASPPR∩A, the cells were rinsed thrice with PBS and fixed in 4% paraformaldehyde for 40 min. Subsequently, the cell membranes were stained with 1 µg mL^−1^ Alexa Fluor 488‐labeled wheat germ agglutinin (WGA) for 90 s on ice. Cover slips were then mounted with DAPI for nuclear visualization and examined under an Olympus FV1000 confocal microscope (Japan). High‐resolution images were captured using a 40x objective lens and processed with the corresponding Olympus FV1000 software.

To evaluate the biodistribution of ASPPR nanocomposites, 1 × 10⁶ 4T1 breast cancer cells were subcutaneously implanted into the right flank of BALB/c mice (6–8 weeks old). Once tumors reached a size of ≈300 mm^3^, ASPPR∩N nanocomposites were administered intravenously. Fluorescence imaging was conducted at 3, 6, 12, 24, and 48‐h post‐injection using an IVIS imaging system (excitation at 530 nm, emission at 570 nm). After imaging, mice were euthanized, and organs (heart, liver, spleen, lungs, kidneys) as well as tumors were harvested for ex vivo fluorescence imaging. Fluorescent intensity measurements were performed in triplicate for reliability and quantified with Spectrum Living Image software.

### Photothermal Properties of ASPPR Nanocomposites: In Vitro and In Vivo Evaluation

To assess the in vitro photothermal properties of ASPPR nanocomposites, their temperature increase was measured in 1000 µL PBS. ASPPR was dispersed at a concentration of 200 µg mL^−1^ and exposed to a 1064 nm laser (1 W cm^−^^2^) for various time intervals.

For in vivo photothermal evaluation, subcutaneous 4T1 tumor‐bearing mice were divided into two groups. When tumor volumes reached ≈150 mm^3^, the animals were intravenously administered either saline or ASPPR nanocomposites (50 mg kg^−1^). After 24 h, the tumors were irradiated with a 1064 nm laser (0.5 W cm^−^^2^) at specified time points. Tumor temperature changes were tracked using an infrared thermal imaging camera to confirm the photothermal effect.

### Western Blot Analysis

To investigate the combined effects of mTOR inhibition and hyperthermia, 4T1 cells (5 × 10⁵) were seeded into six‐well plates and treated with AZD8055 and heat (50 °C) for 24 h. For evaluating the in vitro bioactivity of ASPPR∩A, 4T1 cells were exposed to AZD8055 (2 µM) and an equivalent dose of ASPPR∩A for 2 h, followed by near‐infrared laser irradiation. At designated time points, cells were lysed using RIPA buffer supplemented with protease and phosphatase inhibitors. The resulting protein extracts were subjected to electrophoresis and transferred onto PVDF membranes (Bio‐Rad).

Western blot analysis was conducted using the following antibodies: phosphorylated mTOR (Cell Signaling, #5536S, RRID: AB_10694663, 1/1000), phosphorylated Akt (S473) (Cell Signaling, #4060L, RRID: AB_2315049, 1/2000), phosphorylated S6 (S240/244) (Cell Signaling, #5364L, RRID: AB_10694233, 1/1000), phosphorylated 4EBP1 (Cell Signaling, #2855L, RRID: AB_560835, 1/1000), HLA‐ABC (Proteintech, #15240‐1‐AP, RRID: AB_2115170, 1/2000), B2M (Abcam, #ab218230, RRID: AB_2891403, 1/1000), phosphorylated p38·MAPK (Cell Signaling, #9216S, RRID:AB_3105021, 1/1000), HIF‐1α (Abcam, #ab228649, RRID:AB_2739388,1/1000), HSP70 (Abcam, #5439, RRID:AB_2650529, 1/1000), HSP27 (Cell Signaling, #50353S, RRID:AB_2572328, 1/1000), Cleaved‐caspase‐3 (Cell Signaling, #9664S, RRID:AB_3713233, 1/1000), CHOP (Proteintech, #15204‐1‐AP, RRID:AB_3714679, 1/1000), phosphorylated elF2α (Cell Signaling, #3398T, RRID:AB_2629818, 1/1000), phosphorylated PERK (Proteintech, #15033, RRID:AB_3103093, 1/1000), actin (ZSbio, #TA‐09, RRID:AB_3716396, 1/1000), and α‐tubulin (Abcam, #ab176560, RRID: AB_2737386, 1/2000).

### Assessment of Therapeutic Efficacy in Tumor‐Bearing Mice

The therapeutic potential of ASPPR∩A nanocomposites and their effect on anti‐tumor immune responses were evaluated in BALB/c mice implanted subcutaneously with 5 × 10⁵ 4T1 cells. Once tumors reached ≈80 mm^3^, the mice were randomized into the following treatment groups, with administration every three days via tail vein injection: 1) saline, 2) anti‐PD‐1 antibody (2.5 mg kg^−1^), 3) AZD8055 (2.5 mg kg^−1^), 4) ASPPR nanocarrier (50 mg kg^−1^), 5) ASPPR∩A (containing 2.5 mg kg^−1^ AZD8055 and 50 mg kg^−1^ nanocomposites), 6) AZD8055 + anti‐PD‐1 antibody (2.5 mg kg^−1^ each), and 7) ASPPR∩A + anti‐PD‐1 antibody (2.5 mg kg^−1^ AZD8055 and 2.5 mg kg^−1^ antibody). 24 h post‐injection, tumors were irradiated with a 1064 nm laser (0.5 W cm^−^^2^) for 5 min. On day 24, mice were euthanized, and tumors were harvested for immunohistochemical (IHC) analysis of Ki‐67, CD8, HMGB1, CRT, HSP27, and HSP70. Major organs (heart, liver, spleen, lung, and kidneys) underwent H&E staining for toxicity evaluation.

To assess systemic anti‐tumor effects, a bilateral tumor model was established, implanting 5 × 10⁵ 4T1 cells in both flanks of BALB/c mice. Only the primary tumor (right flank) was treated with ASPPR∩A or control therapies, while the distant tumor (left flank) remained untreated. When the primary tumor reached ≈80 mm^3^, treatments were administered every other day: saline, AZD8055 (2 mg kg^−1^), or ASPPR∩A (equivalent to 2 mg kg^−1^ AZD8055 and 25 mg kg^−1^ nanocomposites), followed by 5 min of laser irradiation (0.5 W cm^−^^2^) on the treated side. Anti‐PD‐1 antibody (2.5 mg kg^−1^) was administered intravenously every six days in the combination therapy group. On day 24, tumors were collected for IHC analysis of CD8, HMGB1, CRT, HSP27, and HSP70. Tumor volumes were measured with calipers every three days and calculated as: Volume = (Tumor length) × (Tumor width)^2^/2.

### Immunohistochemistry and Immunofluorescence Analysis

Tumor samples underwent preparation for immunohistochemistry (IHC) staining, including Hematoxylin and Eosin (H&E), Ki‐67 (Cell Signaling, #12202, RRID: AB_2687824, 1/800), HSP70 (Abcam, #ab5439, RRID: AB_304975, 1/1000), HSP27 (Cell Signaling, #50353S, RRID: AB_2799377, 1/800), HMGB1 (Abcam, #ab79823, RRID: AB_1603374, 1/500), and CD8 (Abcam, #ab217344, RRID: AB_2892040, 1/500) markers. Tumor tissues were fixed in 10% formalin and embedded in paraffin. Sections, 4 µm thick, were mounted on slides for H&E and IHC staining using standardized protocols.

In brief, slides were deparaffinized, treated with 3% hydrogen peroxide to block endogenous peroxidase activity, and subjected to antigen retrieval in EDTA buffer (pH 9.0). After cooling to room temperature, slides were washed with PBS and incubated overnight with primary antibodies. Following additional PBS washes, slides were treated with HRP‐conjugated secondary antibodies at room temperature for 30 min. DAB (3,3′‐diaminobenzidine) was used for chromogenic detection. Finally, slides were rinsed with tap water, counterstained with hematoxylin, dehydrated through an ethanol gradient, and mounted with a medium.

Quantitative analysis, including positive cell density (positive cells per square millimeter) and percentage (positive cells/total nuclei), was performed using ImageJ software.

### Hemolysis Assay

The hemolytic activity of ASPPR nanocomposites was evaluated using freshly collected red blood cells (RBCs) from BALB/c mice. Whole blood was centrifuged at 1500 rpm for 10 min, and the RBC pellet was washed three times with sterile PBS until the supernatant became clear. The RBC suspension (2%, v/v) was incubated with ASPPR nanocomposites at various concentrations (1.25–40 µg mL^−1^) at 37 °C for 2 h. PBS and deionized water were used as negative and positive controls, respectively. After incubation, the samples were centrifuged, and the absorbance of the supernatant at 541 nm was measured using a microplate reader to determine the hemoglobin release. The hemolysis ratio (%) was calculated according to the formula:

(2) $$\mathrm{Hemolysis}\;\mathrm{ratio}=\left(A_{\mathrm{sample}}-A_{\mathrm{negative}}\right)\;/\left(A_{\mathrm{positive}}-A_{\mathrm{negative}}\right)\times 100\%$$

where A_sample_, A_negative_, A_positive_ represent the absorbance of the sample, PBS, and deionized water groups, respectively.

### Masson's Trichrome Staining

Paraffin‐embedded tissue sections (4 µm) were deparaffinized in xylene and rehydrated through a graded ethanol series to distilled water. Nuclei were stained with Weigert's iron hematoxylin for 8 min, followed by thorough rinsing in running tap water for 5 min. Then the sections were stained with Biebrich scarlet–acid fuchsin for 5 min and rinsed briefly in distilled water. Differentiation was performed in phosphomolybdic–phosphotungstic acid solution for 1 min. Without additional rinsing, sections were transferred directly to aniline blue for 2 min to stain collagen. Slides were dehydrated through graded ethanol, cleared in xylene, and mounted with resinous medium.

### TUNEL and CD8 Co‐Staining

Paraffin‐embedded tissue sections (4 µm) were deparaffinized and subjected to antigen retrieval in citrate buffer. TUNEL staining was performed first using a commercial kit according to the manufacturer's protocol. Subsequently, sections were incubated overnight at 4 °C with an anti‐CD8 primary antibody. After washing, a fluorescently‐labeled secondary antibody (Alexa Fluor 594, #A21207, diluted 1:500) was applied and incubated for 1 h at room temperature in the dark. Following the CD8 staining, TUNEL staining was performed using a commercial kit (Beyotime, #C1088). Briefly, the TUNEL reaction mixture was applied to the sections and incubated at 37 °C for 60 min in a humidified, dark chamber. Cell nuclei were stained with DAPI.

### Analysis of Mouse and Human Transcriptome Data

The transcriptome sequencing data of TNBC cells under hyperthermia and mouse fibrosarcoma under PTT treatment were derived from the GEO database (GSE48398, GSE224908). According to previous studies,^[^ ^61^, ^62^, ^63^ ^]^ DAMPs‐related genes were collected, and their information is shown in Table S2 (Supporting Information). Differential expression analysis was performed using the DESeq2^[^ ^64^ ^]^ package (v1.38.3) to normalize raw expression data and model statistical significance under a negative binomial distribution, with significantly differentially expressed genes (DEGs) identified using a Benjamini–Hochberg adjusted p‐value < 0.05 and |log2 fold‐change| > 1; functional annotation of DEGs was subsequently conducted via the clusterProfiler^[^ ^65^ ^]^ package (v4.6.2) for Kyoto Encyclopedia of Genes and Genomes (KEGG) pathway enrichment analysis. Gene Set Variation Analysis^[^ ^66^ ^]^ (GSVA, v1.46.0) was employed to calculate enrichment scores of specific pathways. ImmuCellAI^[^ ^67^ ^]^ for immune infiltration analysis of mouse transcriptome data.

The transcriptome sequencing data of TNBC tumor tissues were derived from the SRA database (SRP157974, PRJNA553096).^[^ ^68^, ^69^ ^]^ Gene expression quantification was performed using kallisto^[^ ^70^ ^]^ (v0.46.1) aligned to the human reference genome GRCh38, followed by xCell‐based^[^ ^71^ ^]^ (v1.1.0) immune infiltration assessment in tumor tissues. TNBC tissues were stratified into AKT‐mTOR high and AKT‐mTOR low subtypes based on AKT‐mTOR signaling pathway activity, using the 25th percentile (1/4) as the cutoff. Gene co‐expression analysis performed with CEMiTool^[^ ^72^ ^]^ identified 1517 significant genes (p < 0.05), which were assigned to 6 distinct modules. Gene Set Enrichment Analysis (GSEA) revealed significant enrichment of modules M1/3/5 in the AKT‐mTOR high subtype and modules M4/6 in the AKT‐mTOR low subtype. Finally, functional annotation based on the KEGG pathway of the module‐specific genes was conducted using clusterProfiler. The stress‐related pathways and IFN‐γ signaling pathway were obtained from the MSigDB database, and their activity levels were assessed using the GSVA package.

### Statistical Analysis

Data analysis was conducted using GraphPad Prism 9 (v9.3.0) and the R package. No data transformation or outlier exclusion was applied. Statistical differences were calculated using independent two‐tailed Student's *t*‐tests or one‐way analysis of variance (ANOVA) followed by Tukey's post‐hoc test for two or multiple group comparisons. Results were presented as mean ± standard deviation (SD). The sample size (n) for each experiment is indicated in the figure legends. The significance level (α) was set at 0.05. P values were denoted as follows: ^*^ *p* < 0.05, ^**^ *p* < 0.01, ^***^ *p* < 0.001, ^****^ *p* < 0.0001. ns indicates no significant difference.

## Conflict of Interest

The authors declare no conflict of interest.

## Author Contributions

Y.Z., J.Y., and X.W. contribute equally to this work. X.L. and J.J. performed in conceptualization. L.L., H.L., X.C., and G.Y. performed in methodology. Y.Z., Y.J., and X.W. performed in investigation. Y.J., X.W., F.Z., X.L., L.T., and L.X. performed in visualization. X.L. and J.J. performed in supervision. Y.Z., Y.J., and X.W. performed in writing—original draft. X.L. and J.J. performed in writing—review and editing.

## Supporting information



Supporting Information

## Data Availability

The data that support the findings of this study are openly available in [GEO, SRA] at [https://www.ncbi.nlm.nih.gov/geo/, https://www.ncbi.nlm.nih.gov/sra/]. These data were derived from the following resources available in the public domain: [GSE48398], [https://www.ncbi.nlm.nih.gov/geo/query/acc.cgi?acc=GSE48398]; [GSE224908], [https://www.ncbi.nlm.nih.gov/geo/query/acc.cgi?acc=GSE224908]; [SRP157974], [https://www.ncbi.nlm.nih.gov/sra/?term=SRP157974]; [PRJNA553096], [https://www.ncbi.nlm.nih.gov/sra/?term=PRJNA553096].
