# Supplementary material for: Combined Photothermal and mTOR‐Targeted Therapy Overcomes Immune Evasion and Enhances Checkpoint Blockade Efficacy in Metastatic Triple‐Negative Breast Cancer
Source: Adv Sci (Weinh). 2025 Nov 19;13(10):e13711. doi: 10.1002/advs.202513711 (PMC12915202; doi:10.1002/advs.202513711)
Supplement: Supplementary file 1 — Supporting Information [file ADVS-13-e13711-s001.docx]

Supplementary Materials for

**Combined Photothermal and mTOR-Targeted Therapy Overcomes Immune Evasion and Enhances Checkpoint Blockade Efficacy in Metastatic Triple-Negative Breast Cancer**

*Yujie Zhao^†^, Jing Yu^†^, Xin Wang^†^, Xu Liu, Fengli Zuo, Tianyue Xu, Leyi Tang, Ling Xiong, Li Li, Huifang Li, Xiaoting Chen, Guang Yang, Jing Jing^*^, Xiaowei Liu^*^*

**This PDF file includes:**

Figure S1. Impact of PTT on AKT-mTOR signaling in TNBC.

Figure S2. AKT-mTOR signaling activity shapes the immunosuppressive tumor microenvironment.

Figure S3. Detailed characterizations of ASPPR.

Figure S4. Heating and cooling curves of ASPPR.

Figure S5. Flow cytometry analysis of Nile Red-labeled ASPPR internalization in TNBC cells and lymphocytes at different incubation times.

Figure S6. Individual tumor growth curves of mice treated with various drugs at the indicated times.

Figure S7. Reversal of PTT-induced PI3K/AKT/mTOR hyperactivation by mTOR inhibition.

Figure S8. Evaluation of the immune regulation of ASPPR∩A nanocomposites.

Figure S9. ICD was assessed through HMGB1, HSP70, and HSP27 IHC staining and H&E staining.

Figure S10. Evaluation of the safety of ASPPR∩A nanocomposites.

Figure S11. Individual growth curves of primary and abscopal tumors in a bilateral 4T1 tumor model under different treatments.

Figure S12. ASPPR∩A-induced ICD primed systemic anti-tumor response.

Table S1. Parameters for photothermal conversion efficiency calculation of ASPPR.

Table S2. DAMPs-related genes.

Table S3. The measurement conditions and parameters for DLS and zeta potential measurements in DMEM culture medium containing 10% fetal bovine serum.


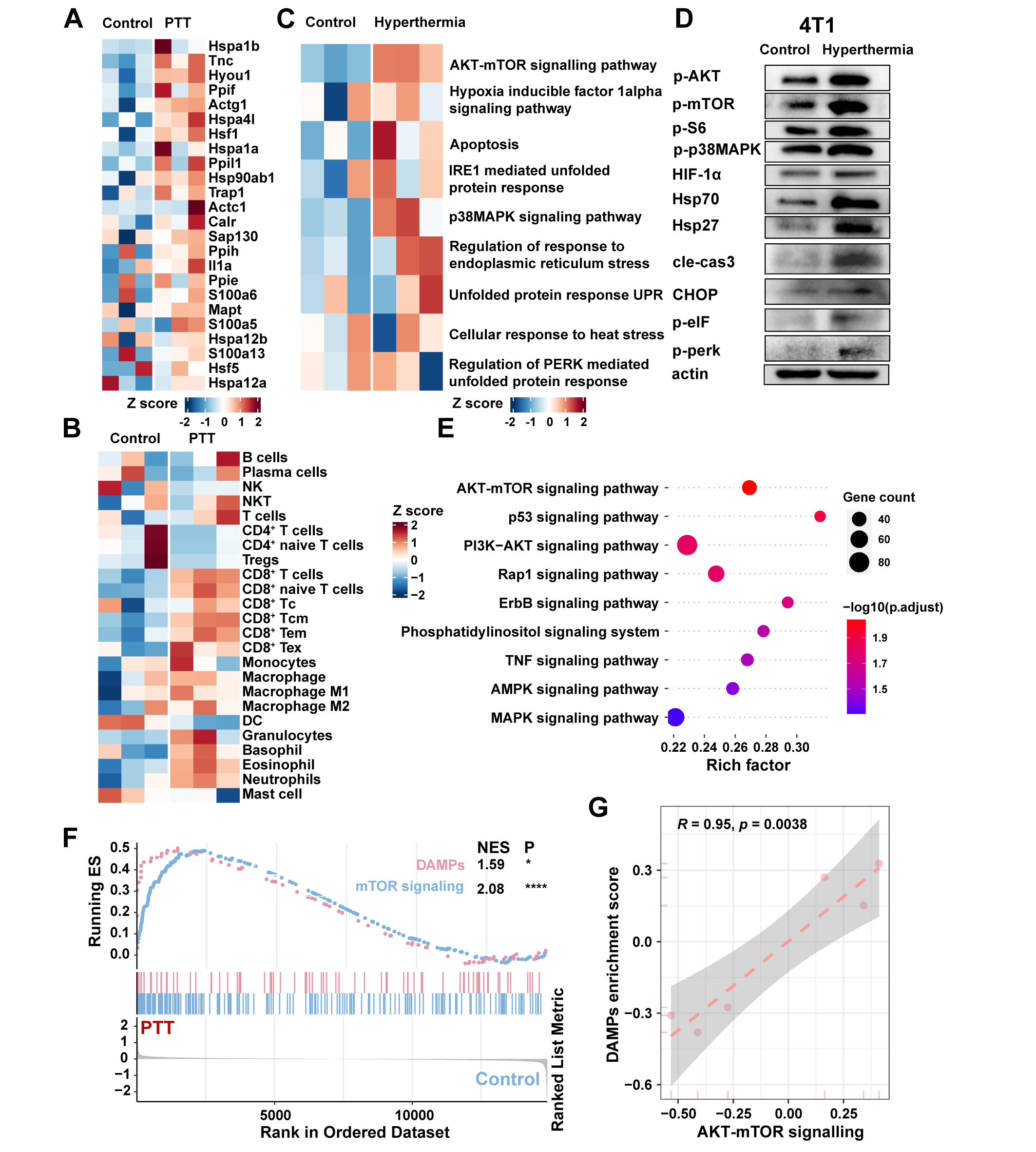


**Figure S1.** Impact of PTT on AKT-mTOR signaling in TNBC. (A) The expression changes of DAMPs genes in the mouse fibrosarcoma tumor tissue before and after PTT treatment (GSE224908). (B) Tumor-infiltrating immune cells were quantified using ImmuCellAI, and differences before and after PTT treatment were visualized using a heatmap (GSE224908). (C) Heatmap shows the changes in stress pathway activity before and after hyperthermia treatment (GSE48398). Pathways are ranked by logFC. (D) Western blot showed the expression of proteins associated with stress pathways in mouse 4T1 cells treated with hyperthermia (50℃). (E) The KEGG pathway enrichment results of DEGs before and after hyperthermia in MDA-MB-468 cells (GSE48398). (F) GSEA plots of the mTOR signaling and DAMPs gene set in the mouse fibrosarcoma dataset (GSE224908). (G) Pearson correlation between DAMPs enrichment score and mTOR signaling pathway (GSE224908). PTT, photothermal therapy; NES, normalized enrichment score; *P < 0.05, ****P < 0.0001.


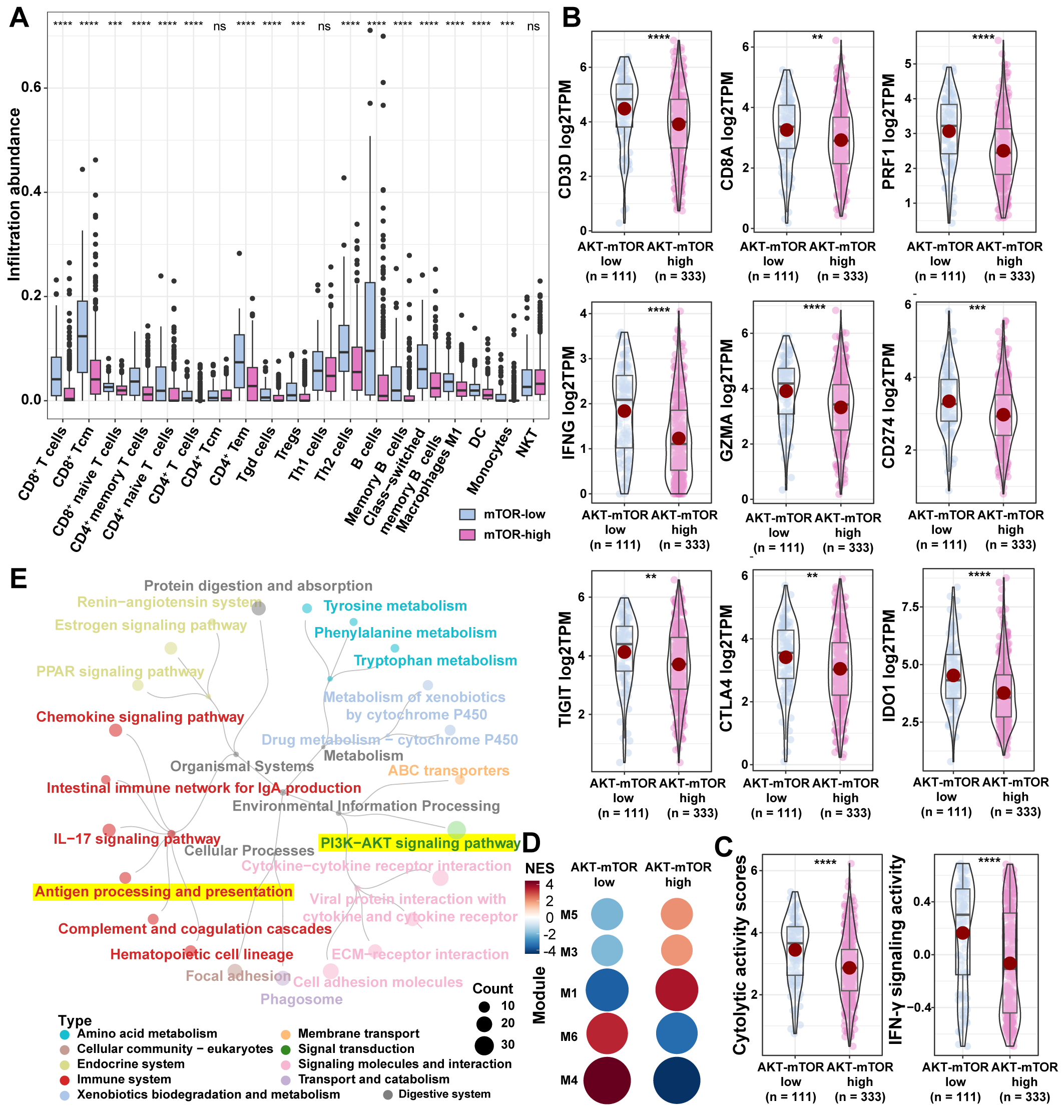


**Figure S2. AKT-mTOR signaling activity shapes the immunosuppressive tumor microenvironment**. (A) Tumor-infiltrating immune cells were quantified using xCell, and differences between AKT-mTOR high and AKT-mTOR low tumors were visualized using a boxplot. Statistical testing was conducted using the Wilcoxon test (SRP157974, PRJNA553096). (B) Box plots illustrate the differences in gene expression between AKT-mTOR high and AKT-mTOR low tumors. Red dots and numerical values indicate mean values. Statistical testing was conducted using the Wilcoxon test (SRP157974, PRJNA553096). (C) Cytolytic activity scores and IFN-γ signaling activity were significantly downregulated in the AKT-mTOR high subtype. Red dots and numerical values indicate mean values. Statistical testing was conducted using the Wilcoxon test (SRP157974, PRJNA553096). (D) GSEA showing the module activity between AKT-mTOR high and AKT-mTOR low tumors (SRP157974, PRJNA553096). Genes within modules (highly interconnected gene clusters) exhibit distinct enrichment patterns based on AKT-mTOR status: genes in modules M1, M3, and M5 are highly enriched in samples with high AKT-mTOR activity; conversely, genes in modules M4 and M6 are highly enriched in samples with low AKT-mTOR activity. (E) The circular dendrogram shows the KEGG pathway enrichment results of M1/3/4/5/6 genes between AKT-mTOR high and AKT-mTOR low tumors. The color of the pathway is marked according to the KEGG Brite hierarchy (SRP157974, PRJNA553096). NES, normalized enrichment score; **P < 0.01, ***P < 0.001, ****P < 0.0001.


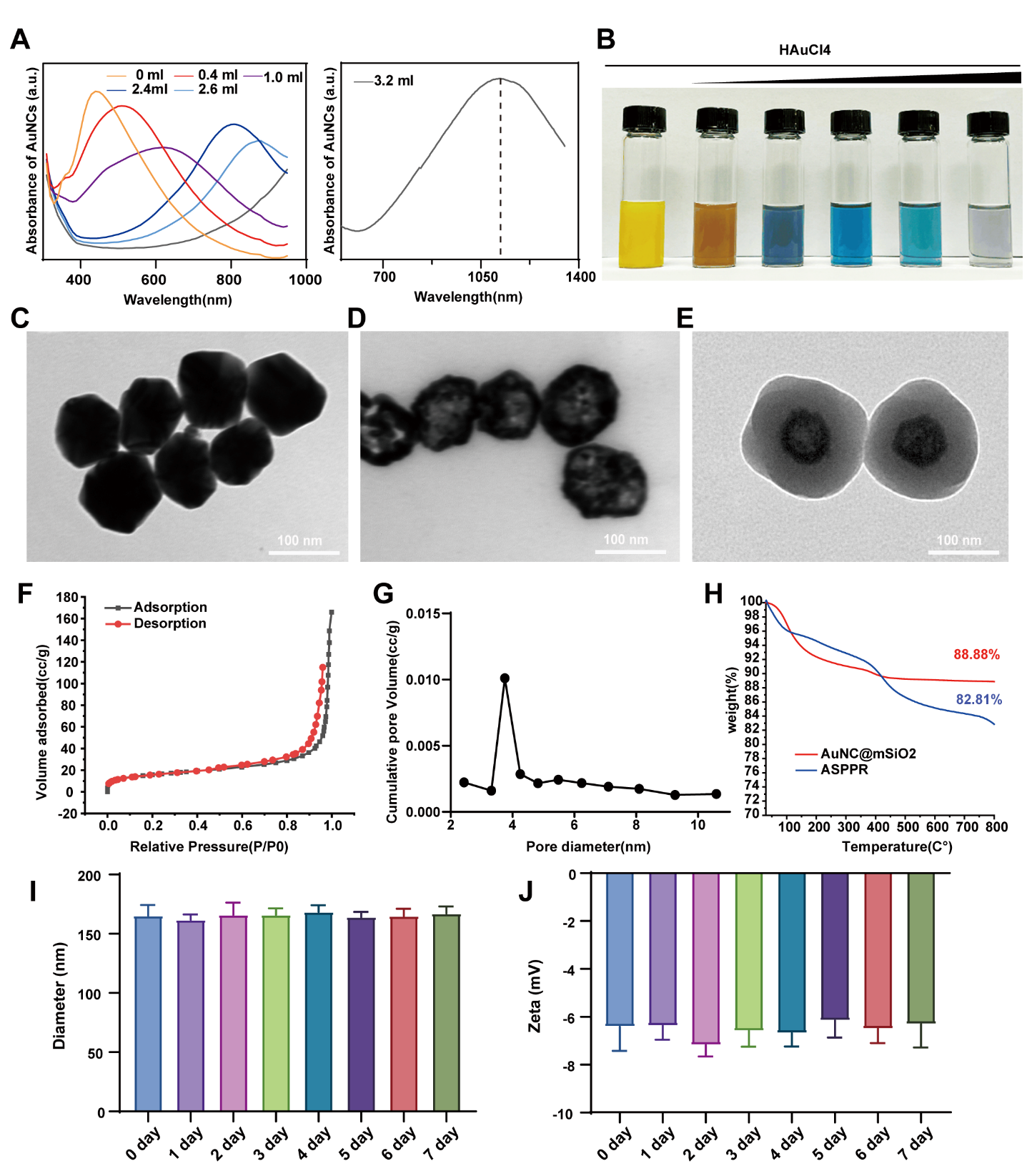


**Figure S3. Detailed characterizations of ASPPR.** (A, B) Absorbance values of gold nanocages (A) and color of gold nanocages solution (B) at different dose of HAuCl_4_. (C-E) TEM images of AgNC (C), AuNC (D), and AuNC@SiO_2_ (E). Scale bar 100 nm. (F, G) Nitrogen adsorption-desorption isotherms of AuNC@mSiO_2_ (F) and pore size distribution of AuNC@mSiO_2_ (G). (H) TGA of AuNC@mSiO_2_ and ASPPR. (I) Hydrodynamic diameter and (J) zeta potential of ASPPR nanoparticles measured by dynamic light scattering over 0-7 days in 10% FBS medium. The results were presented as mean ± SD (n=3).


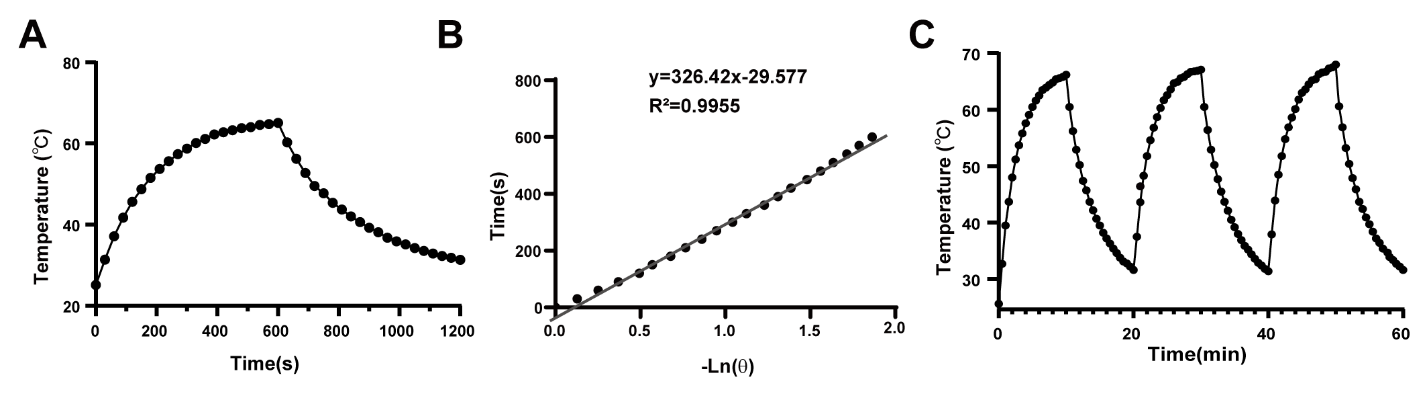


**Figure S4. Heating and cooling curves of ASPPR.** (A) ASPPR was irradiated by a 1064 nm laser at 1 W/cm^2^ for 10 min, and then the laser was switched off and the cooling rate was recorded. (B) Linear fit of time versus the negative natural logarithm of the temperature increment for ASPPR cooling rate. (C) Light resistance test of ASPPR.


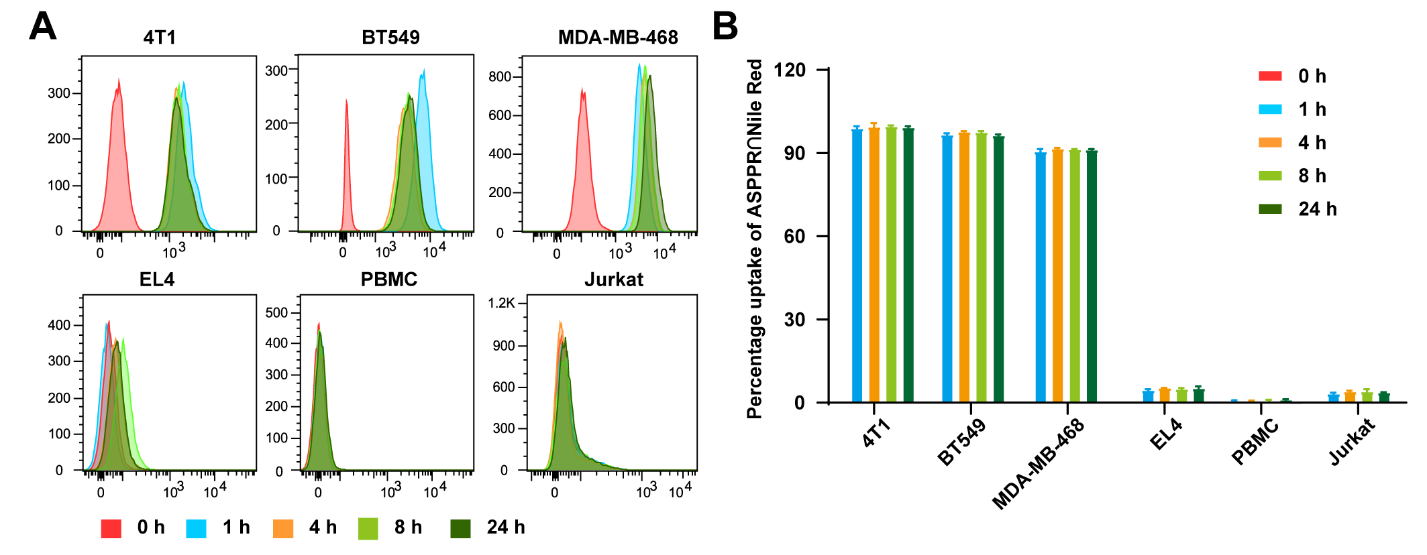


**Figure S5.** **Flow cytometry analysis of Nile Red-labeled ASPPR internalization in TNBC cells and lymphocytes at different incubation times.** (A, B) TNBC cells (4T1, BT549, MDA-MB-468) and lymphocytes (EL4, human PBMC, Jurkat) were incubated with Nile Red-labeled ASPPR for 1, 4, 8, and 24 h. The internalizations were determined by flow cytometer (A) and the internalizations were quantified (B). The data presented mean ± SD (n = 3).


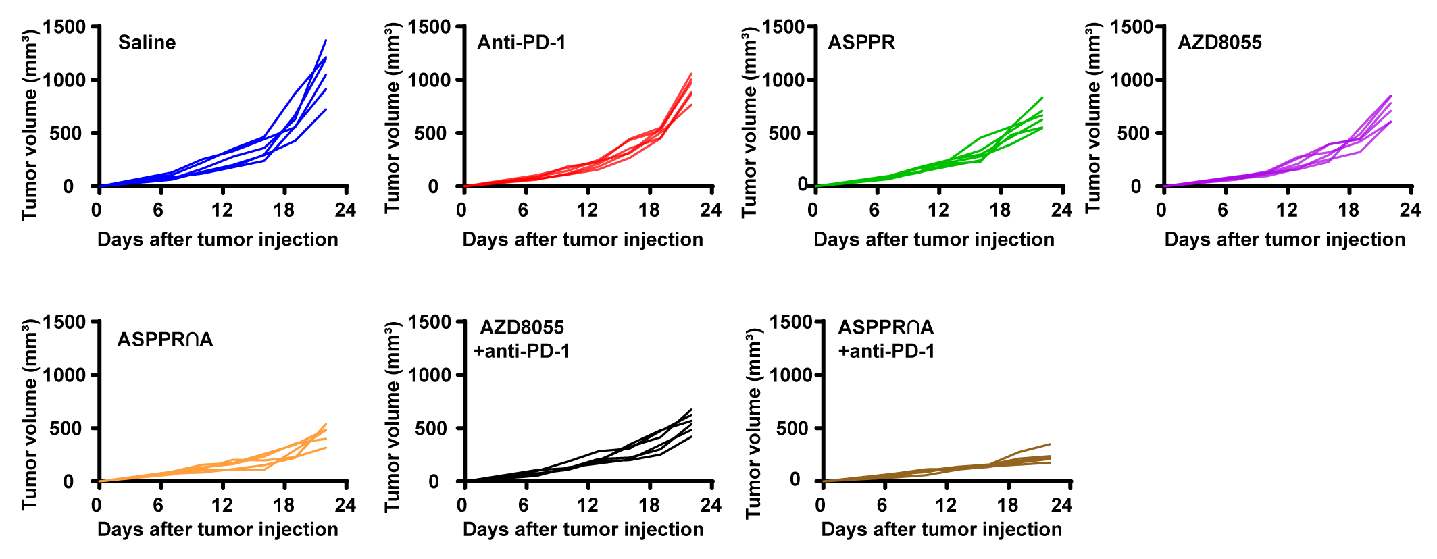


**Figure S6. Individual tumor growth curves of mice treated with various drugs at the indicated times.** BALB/c mice with subcutaneous 4T1 tumors were randomly assigned to seven groups (n = 6 per group): (1) saline, (2) anti-PD-1, (3) AZD8055, (4) ASPPR, (5) ASPPR∩A, (6) AZD8055 + anti-PD-1, and (7) ASPPR∩A + anti-PD-1. AZD8055, ASPPR, and ASPPR∩A were administered intravenously every 3 days. Groups (4), (5), and (7) received 1064 nm laser irradiation (0.5 W/cm², 5 min) 24 h post-injection. Anti-PD-1 antibody was administered every 6 days. Tumor volumes were monitored throughout the treatment to assess therapeutic efficacy.


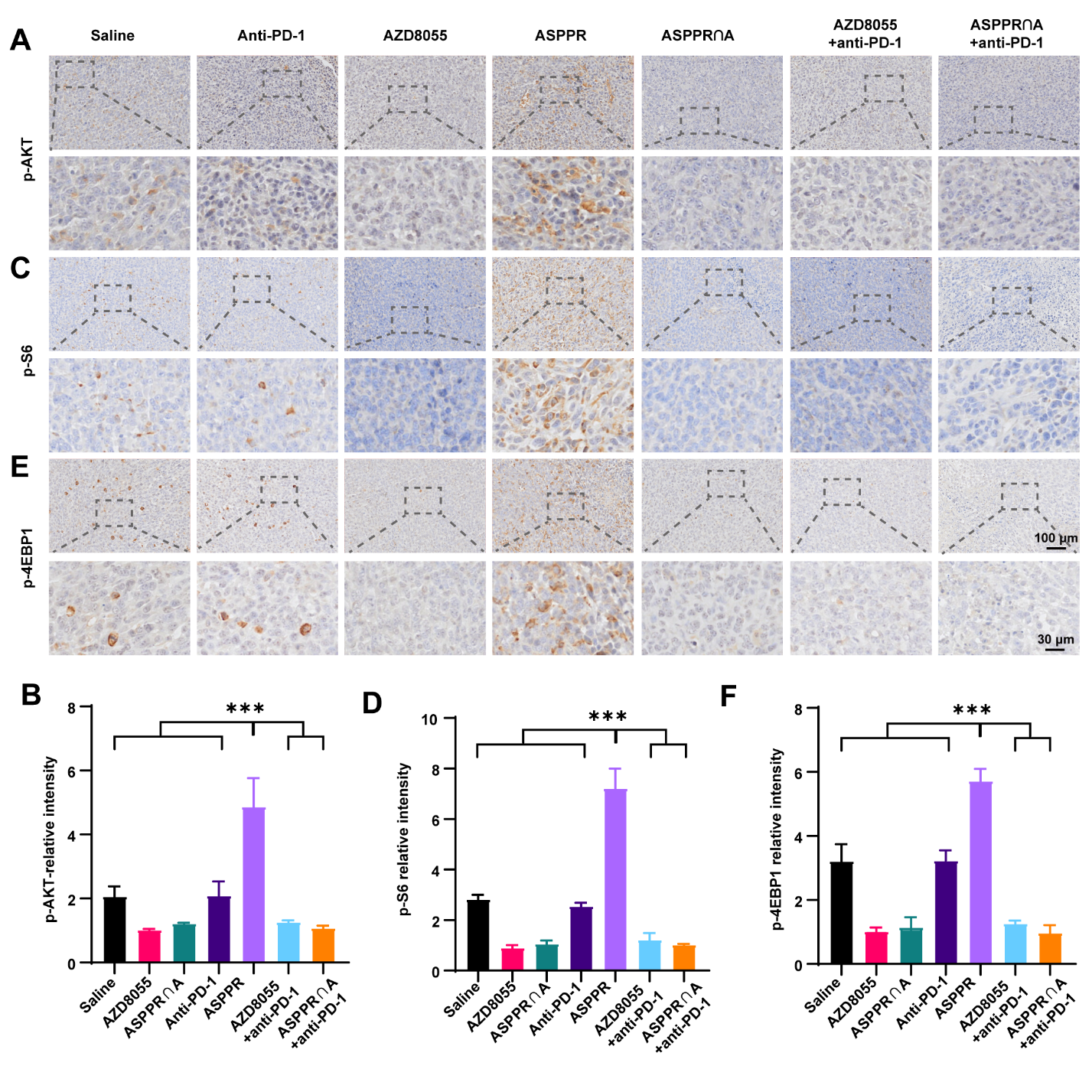


**Figure S7. Reversal of PTT-induced PI3K/AKT/mTOR hyperactivation by mTOR inhibition.** (A, C, E) The expression of p-AKT (A), p-S6 (C) and p-4EBP1 (E) was visualized by IHC staining, scale bar 100 μm. (B, D, F) The relative intensity of p-AKT (B), p-S6 (D), and p-4EBP1 (F) in each group was quantified. The quantitative results were presented as mean ± SD (n=3), *P < 0.05, **P < 0.01, and ***P < 0.001, analyzed by ANOVA.


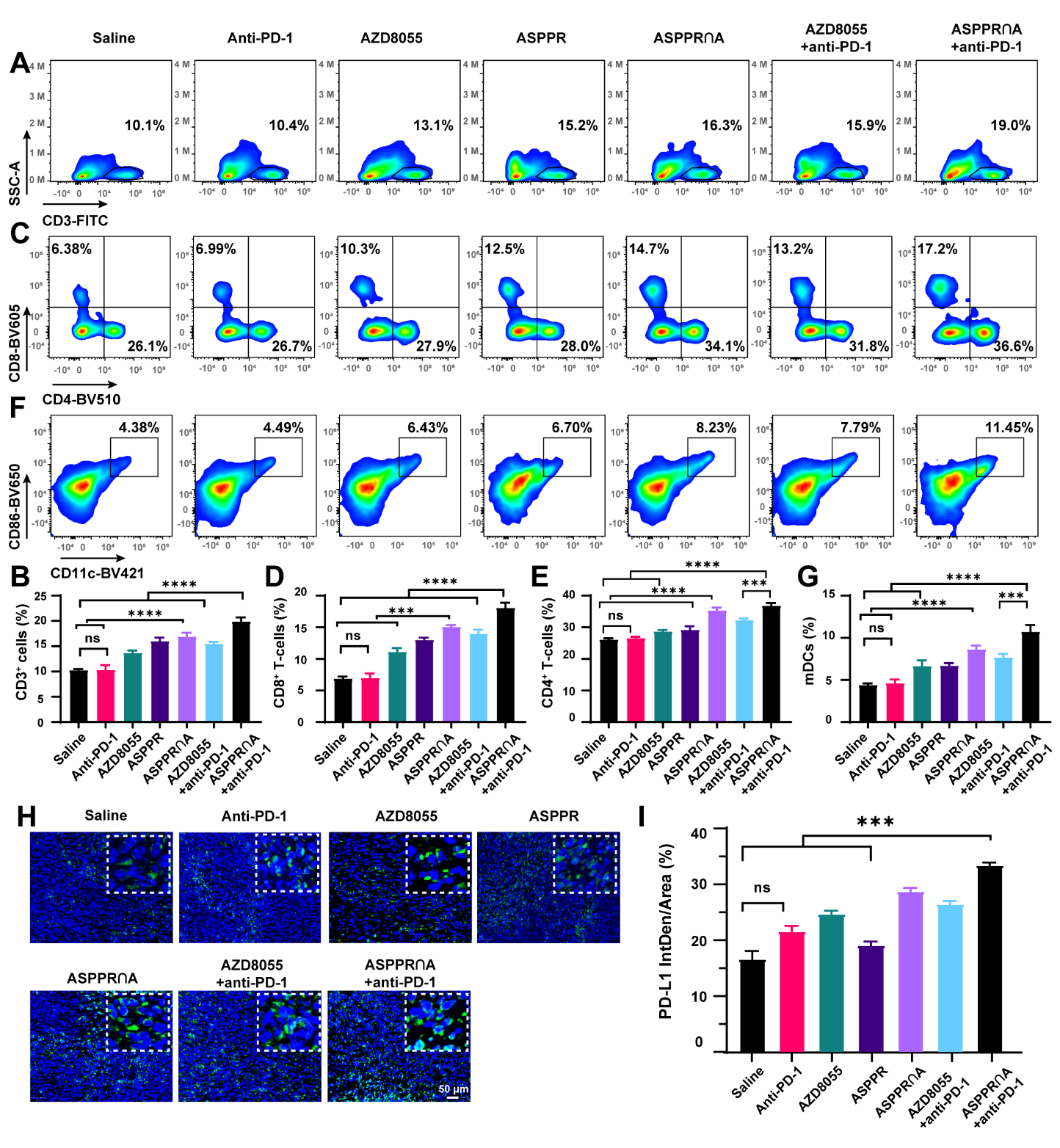


**Figure S8. Evaluation of the immune regulation of ASPPR∩A nanocomposites.** (A, C, F) Representative flow cytometry plots showing gating of CD3⁺ T cells, CD3⁺CD8⁺ cytotoxic T lymphocytes (CTLs), CD3⁺CD4⁺ T helper cells, and mature dendritic cells (mDCs, CD11c⁺CD86⁺) in tumor tissues. (B, D, E, G) Quantification of CD3⁺ T cells (B), CD8⁺ T cells (D), CD4⁺ T cells (E), and mDCs (G) among different treatment groups. Data were presented as mean ± SD, n = 3. (H) Representative immunofluorescence images of PD-L1 (green) in 4T1 tumor sections from mice treated with saline, anti–PD-1, AZD8055, ASPPR, ASPPR∩A, AZD8055 + anti–PD-1, or ASPPR∩A + anti–PD-1. Nuclei were stained with DAPI (blue). Scale bar, 50 μm. (I) Quantification of PD-L1 expression in each group. The quantitative results were presented as mean ± SD (n=3). ns, not significant, *P < 0.05, **P < 0.01, and ***P < 0.001, analyzed by ANOVA.


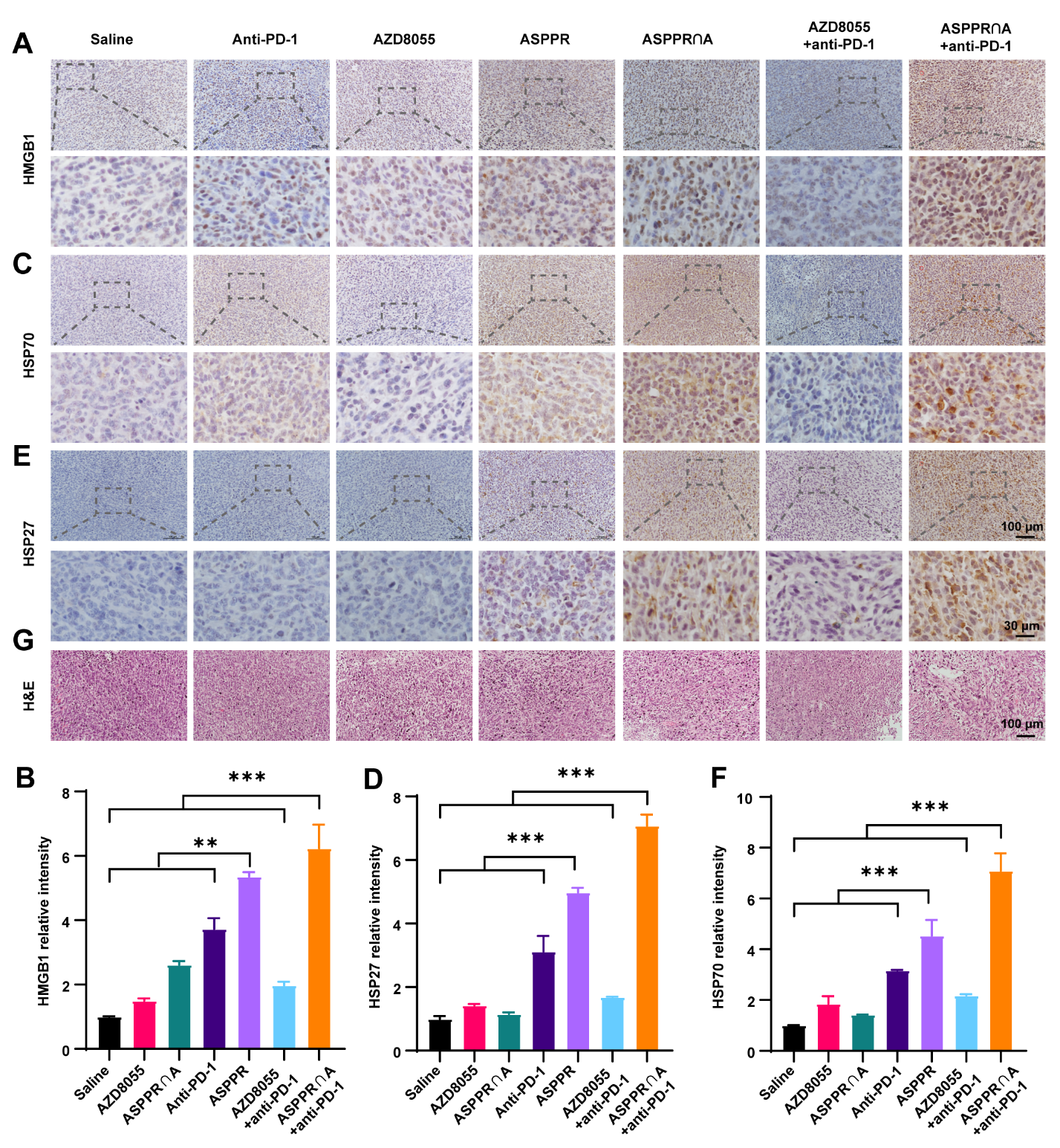


**Figure S9. ICD was assessed through HMGB1, HSP70, and HSP27 IHC staining and H&E staining.** (A, C, E) ICD was visualized by HMGB1 (A), HSP70 (C), and HSP27 (E) IHC staining, scale bar 100 μm. (B, D, F) The relative intensity of HMGB1(B), HSP70 (D), and HSP27 (F) in each group was quantified. The quantitative results were presented as mean ± SD (n=3), *P < 0.05, **P < 0.01, and ***P < 0.001, analyzed by ANOVA. (G) H&E staining of the tumor tissues from tumor-bearing mice treated with the indicated formulations. Scale bar 100 μm.


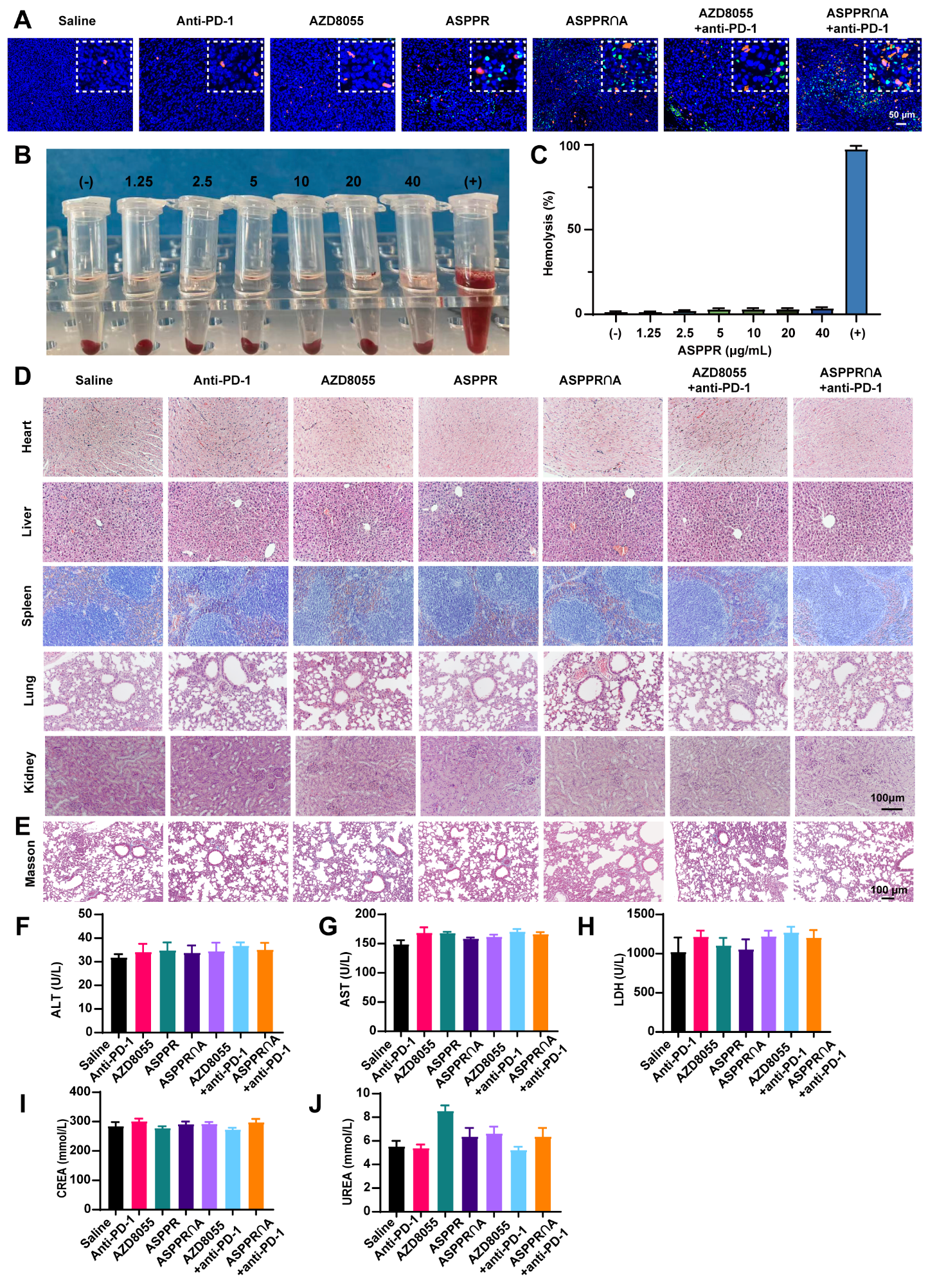


**Figure S10. Evaluation of the safety of ASPPR∩A nanocomposites.** (A) Immunofluorescence co-staining of CD8 (red) and TUNEL (green) in tumor sections from different treatment groups. Nuclei were stained with DAPI (blue). Scale bar, 50 μm. (B) Representative images of red blood cells incubated with ASPPR at different concentrations (1.25-40 μg/mL), along with negative (PBS) and positive (deionized water) controls. (C) Quantitative hemolysis analysis, Data were presented as mean ± SD. n=3. (D) H&E staining of the main organs (heart, liver, spleen, lung, and kidney) from tumor-bearing mice treated with indicated formulations. Scale bar 100 μm. (E) Representative lung sections from different treatment groups stained with Masson’s trichrome for fibrosis assessment. Scale bar, 100 μm. (F-J) Serum biochemical values of mice at day 24 for saline, AZD8055, anti-PD-1, ASPPR + laser, AZD8055 + anti-PD-1, ASPPR∩A + laser, and ASPPR∩A + laser + anti-PD-1 treated groups. Data presented as mean ± SD (n = 3). Note: ALT, aspartate transaminase; AST, aspartate aminotransferase; LDH, lactate dehydrogenase.


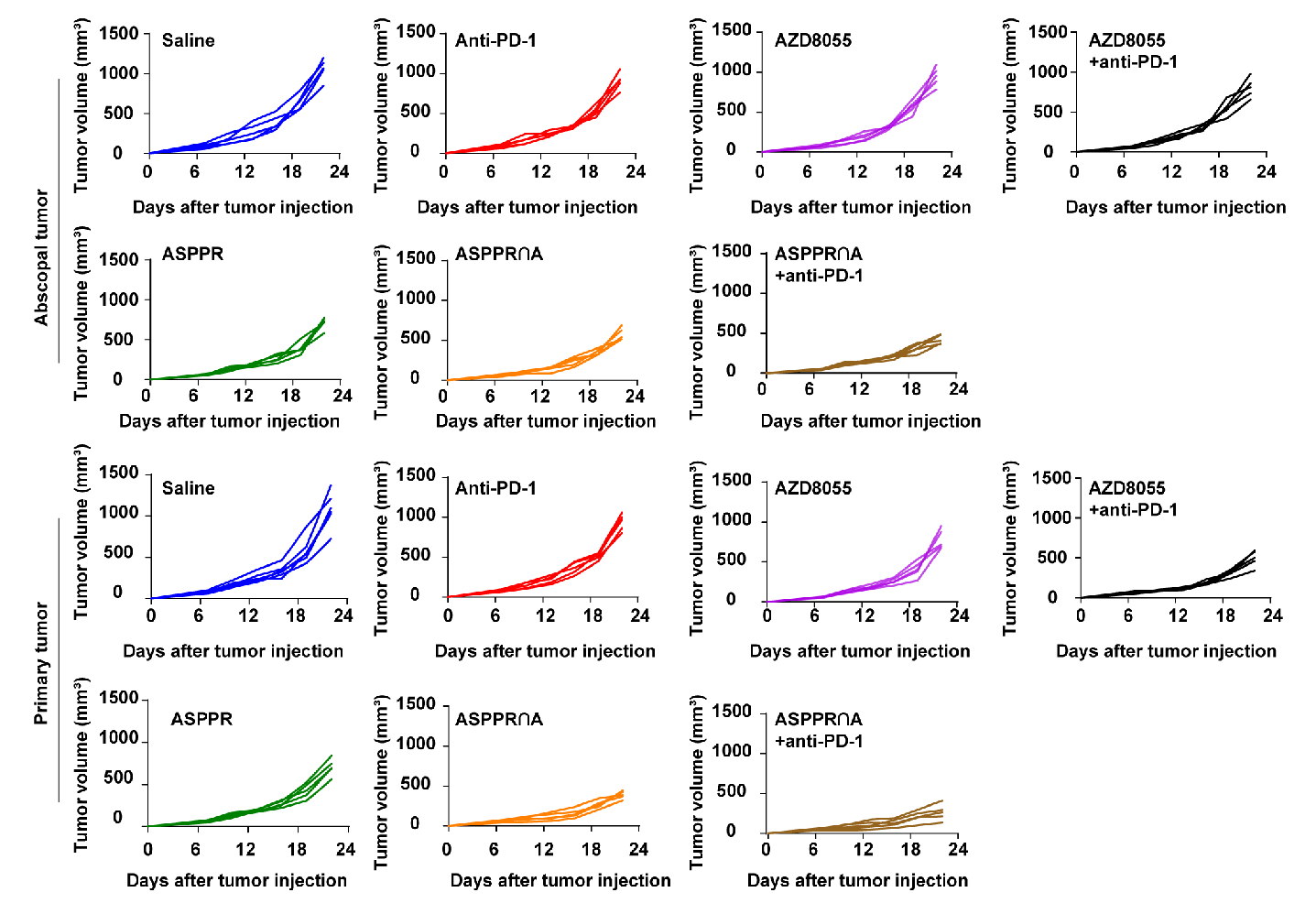


**Figure S11. Individual growth curves of primary and abscopal tumors in a bilateral 4T1 tumor model under different treatments.** BALB/c mice were subcutaneously inoculated with 4T1 cells on both flanks to establish bilateral tumors. Treatments were locally applied to the primary tumor (right flank) when it reached ~80 mm³, while the abscopal tumor (left flank) remained untreated. Mice were randomly divided into seven groups (n = 5 per group): (1) saline, (2) anti-PD-1, (3) AZD8055, (4) ASPPR, (5) ASPPR∩A, (6) AZD8055 + anti-PD-1, and (7) ASPPR∩A + anti-PD-1. Tumor volumes were measured every 3 days for both primary and distant sites to evaluate local and systemic therapeutic responses.


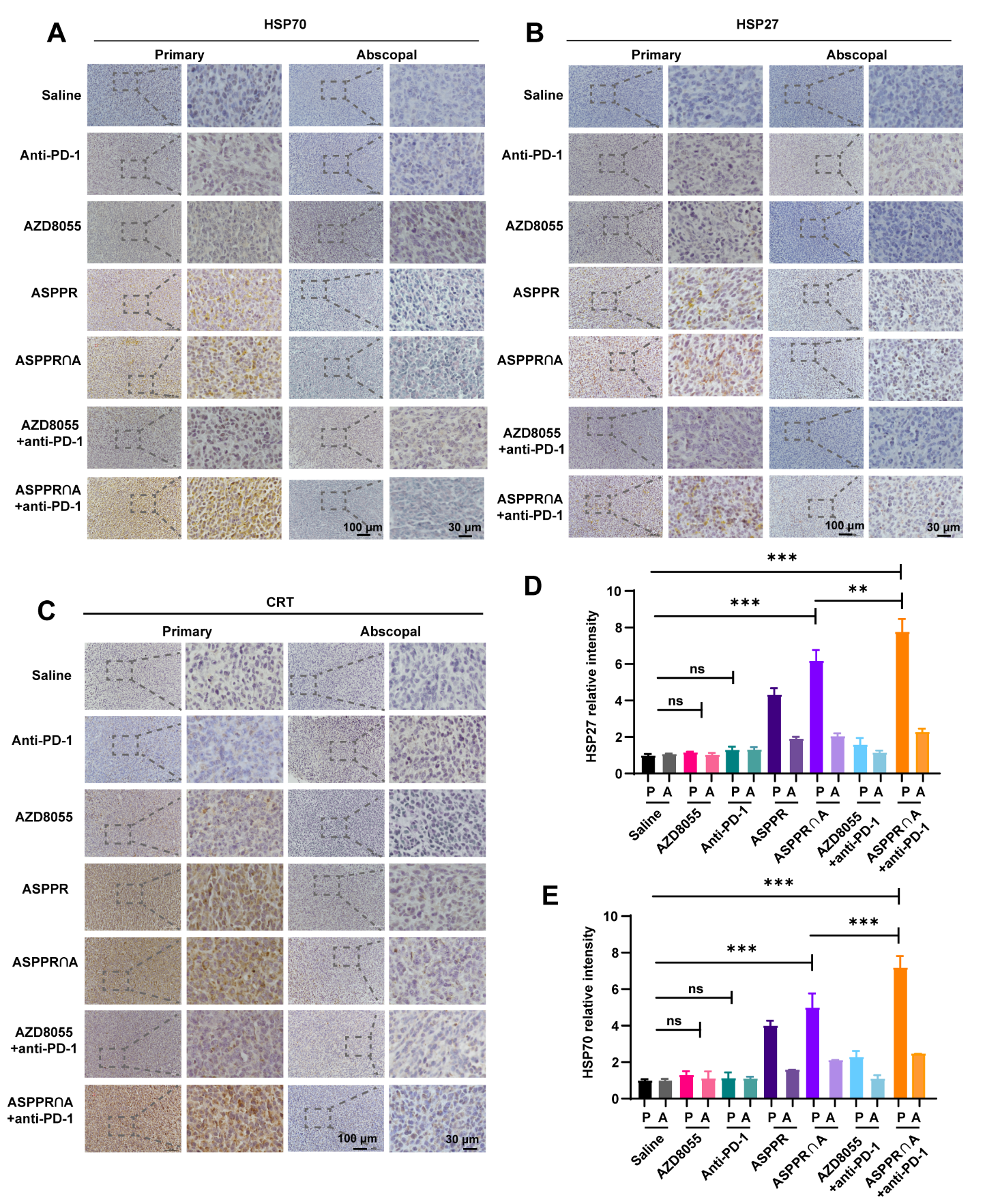


**Figure S12. ASPPR∩A-induced ICD primed systemic anti-tumor response.** (A-C) ICD was visualized by HSP70 (A), HSP27 (B), and CRT (C) IHC staining, scale bar 100 μm. (D, E) The relative intensity of HSP27 (D), and HSP70 (E) in each group was quantified. P, primary tumor. A, abscopal tumor. The quantitative results were presented as mean ± SD (n=3). ns, not significant, *P < 0.05, **P < 0.01, and ***P < 0.001, analyzed by ANOVA.

**Table S1. Parameters for photothermal conversion efficiency calculation of ASPPR.**

|  | ASPPR |
| --- | --- |
| τ_s_ | 326.42 |
| hs | 0.013 |
| OD | 1.000 |
| 10^-A^ | 0.100 |
| I (W) | 1.00 |
| ∆T (K) | 40 |
| T_max_ (K) | 65.1 |
| ƞ | 0.3988 |
| ƞ/% | 39.88% |

η=[hs(T_max_-T_sur_)-Q_Dis_]/[I(1-10^-A^)]

hs=m_D_C_D_/τ_s_

Q_Dis_=hs(T_h2O_-T_sur_)

**Table S2. DAMPs-related genes.**

| TRAP1 | CIRBP | HSF4 | HSPA6 | HYOU1 | PPIC | RANBP2 | S100A5 |
| --- | --- | --- | --- | --- | --- | --- | --- |
| ACTA1 | CRYAA | HSF5 | HSPA7 | IL1A | PPID | ROCK1 | S100A6 |
| ACTA2 | FGA | HSP90AB1 | HSPA8 | IL33 | PPIE | RPS3 | S100A7L2 |
| ACTB | FGB | HSPA12A | HSPA9 | MAPT | PPIF | S100A1 | S100B |
| ACTBL2 | FGG | HSPA12B | HSPB2 | NKTR | PPIG | S100A10 | S100G |
| ACTC1 | HMGB1 | HSPA13 | HSPB3 | NMI | PPIH | S100A12 | S100Z |
| ACTG1 | HMGB2 | HSPA14 | HSPB6 | ODF1 | PPIL1 | S100A13 | SAP130 |
| ACTG2 | HMGN1 | HSPA1B | HSPB7 | PANX1 | PPIL2 | S100A16 | TNC |
| BCL2 | HSBP1 | HSPA2 | HSPB8 | PARK7 | PPIL4 | S100A2 |  |
| CALR | HSF1 | HSPA4L | HSPB9 | PPIA | PPIL6 | S100A3 |  |
| CENPA | HSF2 | HSPA5 | HSPH1 | PPIB | PPWD1 | S100A4 |  |

**Table S3. The measurement conditions and parameters for DLS and zeta potential measurements in DMEM culture medium containing 10% fetal bovine serum.**

| Dispersant refractive indices | 1.3330 |
| --- | --- |
| Sample dilutions | 50 |
| Dispersion media | DMEM culture medium containing10% fetal bovine serum |
| Scattering angles (°) | 90 |
